# Extracellular lipidosomes containing lipid droplets and mitochondria are released during melanoma cell division

**DOI:** 10.1186/s12964-024-01471-7

**Published:** 2024-01-19

**Authors:** Jana Karbanová, Ilker A. Deniz, Michaela Wilsch-Bräuninger, Rita Alexandra de Sousa Couto, Christine A. Fargeas, Mark F. Santos, Aurelio Lorico, Denis Corbeil

**Affiliations:** 1 Biotechnology Center (BIOTEC) and Center for Molecular and Cellular Bioengineering, Technische Universität Dresden, Tatzberg 47-49, Dresden, 01307 Germany; 2 Tissue Engineering Laboratories, Medizinische Fakultät der Technischen Universität Dresden, Fetscherstr. 74, Dresden, 01307 Germany; 3 Max Planck Institute of Molecular Cell Biology and Genetics, Pfotenhauerstrasse 108, Dresden, 01307 Germany; 4 Escola Superior de Biotecnologia, Universidade Católica Portuguesa, Rua de Diogo Botelho 1327, Porto, 4169-005 Portugal; 5 College of Osteopathic Medicine, Touro University Nevada, 874 American Pacific Drive, Henderson, NV 89014 USA; 6 Tissue Engineering Laboratories, Biotechnology Center, Technische Universität Dresden, Tatzberg 47-49, Dresden, 01307 Germany

**Keywords:** Cell division, Extracellular vesicle, Lipid droplet, Melanoma, Mitochondrion, Prominin-1

## Abstract

**Background:**

The incidence of melanoma is increasing worldwide. Since metastatic melanoma is highly aggressive, it is important to decipher all the biological aspects of melanoma cells. In this context, we have previously shown that metastatic FEMX-I melanoma cells release small (< 150 nm) extracellular vesicles (EVs) known as exosomes and ectosomes containing the stem (and cancer stem) cell antigenic marker CD133. EVs play an important role in intercellular communication, which could have a micro-environmental impact on surrounding tissues.

**Results:**

We report here a new type of large CD133^+^ EVs released by FEMX-I cells. Their sizes range from 2 to 6 µm and they contain lipid droplets and mitochondria. Real-time video microscopy revealed that these EVs originate from the lipid droplet-enriched cell extremities that did not completely retract during the cell division process. Once released, they can be taken up by other cells. Silencing CD133 significantly affected the cellular distribution of lipid droplets, with a re-localization around the nuclear compartment. As a result, the formation of large EVs containing lipid droplets was severely compromised.

**Conclusion:**

Given the biochemical effect of lipid droplets and mitochondria and/or their complexes on cell metabolism, the release and uptake of these new large CD133^+^ EVs from dividing aggressive melanoma cells can influence both donor and recipient cells, and therefore impact melanoma growth and dissemination.

**Supplementary Information:**

The online version contains supplementary material available at 10.1186/s12964-024-01471-7.

## Background

Melanoma is a very aggressive disease responsible for the vast majority of deaths from skin cancer [1]. Melanoma is often incurable once the cancer cells have spread from the primary site to other tissues or organs. It has a prevalence of early lymph node metastases, which can originate from thin primary tumors [2–4]. Any intervention that can limit the propagation of melanoma cells or eradicate them at an early stage can have a therapeutic application. Potentially metastatic cells may constitute a minority of the total population of melanoma cells that needs to be targeted to prevent their dissemination.

In search of target molecules potentially having an impact on melanoma cells, we came across the cholesterol-binding membrane protein CD133, a widely used marker for stem cells and cancer-initiating cells [5–8] (reviewed in Refs [9–11]). Our investigations have shown that silencing CD133 in FEMX-I cells, an aggressive melanoma cell line derived from a lymph node metastasis of a patient with malignant melanoma [12, 13], negatively affected their growth and motility as well as their potential to metastasize upon injection in a murine model [14]. In agreement with these observations, a correlation between CD133 expression in childhood malignant melanoma and lymph node and/or visceral metastasis was found [15]. Subpopulations of CD133^+^ melanoma were also shown to contribute to perivascular niche morphogenesis and tumorigenicity through vasculogenic mimicry [16]. Thus, the cell surface protein CD133 could be a potential candidate as a target molecule for the therapy of lethal skin cancer [9, 17].

Moreover, a relationship between CD133 and Wnt/β-catenin signaling pathway, and their effect on lipid droplets was drawn up, as CD133-deficient melanoma cells showed a partial reduction in lipid droplets [14, 18]. The suppression of CD133 prevented the nuclear localization of β-catenin and reduced Wnt pathway signaling through T-cell factor/lymphoid enhancing factor transcription factors. Similar observations were also made with colorectal cancer stem cells, suggesting a broader implication for carcinogenesis [19] (reviewed in Refs [20, 21]). The relationship between CD133 and lipid droplets needs further research to fully understand its importance and potential consequences on cancer stem cell properties. Lipids play essential cellular roles as energy storage and/or signaling molecules, in addition to a structural function in the organization and dynamics of membranes. Any alteration in their regulation can promote tumorigenesis and the metastatic properties of cancer cells [22]. In such context, it was recently shown that lipid droplets are at the crossroad of oxidative metabolism and lipid(s) regulation that can disrupt cell cycle progression and slow melanoma growth in vivo [23]. Thus, melanoma cells with enhanced lipid droplet capacity are at a metabolic advantage.

In addition to its direct influence on cell properties, we found that CD133 was associated with small extracellular vesicles (EVs) such as exosomes and/or ectosomes released by FEMX-I cells [18, 24]. The release of CD133^+^ EVs has been associated with the differentiation of stem and progenitor cells [25]. EVs are of great interest because of their role in intercellular communication as carriers of biomaterials which, when taken up by other cells, can influence their fate [26–29]. This is particularly important in the field of oncology, where EVs derived from cancer cells can transform the cellular environment [30, 31]. Here, we described a new type of CD133^+^ EVs containing lipid droplets that are released during melanoma cell division and/or cell migration. These large EVs contain not only lipid droplets but also mitochondria. It is well established that these two organelles interact in synergy, the mitochondria being the energy factory and the lipid droplets the energy reservoir [32–34]. In addition, lipid droplet-associated mitochondria have increased bioenergetic capacity, reduced β-oxidation capacity and support lipid droplet expansion [35]. The release of these large EVs can therefore have an impact not only on the metabolism of the donor cancer cells, but also on the recipient cells, and thus influences the cancer cell microenvironment. Altogether, we found that aggressive melanoma cells release large CD133^+^ EVs containing lipid droplets and mitochondria, and that CD133 itself can contribute to this newly discovered cellular process.

## Methods

### Reagents and antibodies

Poly-L-lysine (catalog number (#) P4707) was obtained from Sigma-Aldrich (St. Louis, MO), while human fibronectin (#356008) was purchased from Corning Inc. (Corning, NY). The latter was dissolved in distilled water at a final concentration of 50 µg/mL. Carbonyl cyanide m-chlorophenyl hydrazine (CCCP) obtained from Sigma-Aldrich (#C2759) was dissolved in dimethyl sulfoxide (DMSO) at a stock solution of 10 mM. BODIPY™ 493/503 (4,4-Difluoro-1,3,5,7,8-Pentamethyl-4-Bora-3a,4a-Diaza-*s*-Indacen, #D3922) and MitoTracker™ Red CMXRos (#M7512) were purchased from Thermo Fisher Scientific. BODIPY™ 493/503 was resuspended in ethanol at a stock solution of 1 mg/ml and used at a final concentration of 7.6 µM in phosphate-buffered saline (PBS). LipidSpot™ 488/610 Lipid Droplet Stains (#70065/#70069) and CF®488A, CF®640R or CF®555-conjugated wheat germ agglutinin (WGA; #29022, #29026 and #29076, respectively) as well as MitoView™ Fix 640 (#70082), were obtained from Biotium Inc. (Fremont, CA). In some experiments Rhodamine-conjugated WGA (#RL-1022) from Vector Laboratories (Burlingame, CA) was used. Alexa Fluor®546 phalloidin (#A22283) was from Thermo Fisher Scientific.

The primary antibodies used in this study were the following: anti-adipophilin/perilipin 2 monoclonal antibody (clone AP125, #651102; dilution 1:40) from PROGEN Biotechnik GmbH (Heidelberg, Germany); anti-CD133 monoclonal antibodies CD133/1 (clone W6B3C1, #130–092-395; 1:50 and clone AC133, #130–090-422; 1:50) and CD133/2 (293C3, #130–090-851; 1:50) all from Miltenyi Biotec GmbH (Bergisch Gladbach, Germany) and our own monoclonal antibody 80B258 (1:2000) [36]; anti-Alix monoclonal antibody 3A9 (#MCA2493; 1:100) from AbD Serotec/Bio-Rad Laboratories GmbH (Feldkirchen, Germany) and anti-α–tubulin monoclonal antibody DM1A (#T6199; 1:200) from Sigma-Aldrich; anti-Vimentin monoclonal antibody V9 (#sc-6260; 1:50), anti-Nestin monoclonal antibody 10C2 (#MAB5326; 1:100) and anti-mitochondrial antigen monoclonal antibody (113–1, #MU213-5UC; 1:50) were purchased from Santa Cruz Biotechnology Inc. (Dallas, TX), MilliporeSigma (Temecula, CA) and BioGenex (Fremont, CA), respectively.

Various anti-integrin (ITG) monoclonal antibodies were used either for flow cytometry or immunocytochemistry according to manufacturer's recommendations. For flow cytometry, the anti-CD29 (ITG β1) antibody conjugated with allophycocyanin (APC) (TS2/16, #303007), CD49b (ITG α4)-APC (P1E6-C5, #359309), CD49e (ITG α5)-APC (NKI-SAM-1, #328011), CD51 (ITG αV) coupled to phycoerythrin (PE) (NK1-M9, #327909), CD51/61 (ITG αV/β3)-APC (923C6, #304415), CD61 (ITG β3)-APC (VI-Pl2, #336411) and CD104 (ITG β4)-PE (58XB4, #327807) were all obtained from BioLegend (San Diego, CA), while anti-CD11b (ITG αM)-PE (M1/70.15.11.5, #130–091-240), CD49d (ITG α4)-APC (MZ18-24A9, #130–093-281) were from Miltenyi Biotec. The anti-CD11c (ITG αX)-APC (Bly-6, #559887), CD18 (ITG β2) (6.7, #555922), CD41a (ITG α2b)-APC (HIP8, #559777), CD49f (ITG α6) (GoH3, #555734) were from BD Biosciences (Heidelberg, Germany) and CD103 (ITG AE or αE)-APC (B-ly7, #17–1038-42) from Thermo Fisher Scientific. Isotype controls IgG1–PE/-APC (MOPC-21, #555749/555751), IgG2a-PE (MOPC-173, #400214) and IgG2b-PE/-APC (IS6-11E5.11, #130–099-875, 130–122-976) were from BD Biosciences, BioLegend and Miltenyi Biotec, respectively. For immunocytochemistry, unconjugated antibody CD29 (TS2/16, #303002; 1:50), CD49b (P1E6-C5, #359301; 1:50), CD49e (NKI-SAM-1, #328002; 1:50) and CD51/61 (23C6, #304402; 1:50) or PE-conjugated CD51 (NK1-M9; 1:50) were all purchased from BioLegend, while CD49f (GoH3, #555734; 1:50) and CD61 (VI-Pl2, #555752; 1:50) were obtained from BD Biosciences.

For flow cytometry, when the primary antibody was not directly conjugated with a given fluorochrome, we used PE-conjugated F(ab’)2 donkey anti-rat IgG (#12–4822-82; 1:100) or APC-conjugated F(ab’)2 goat anti-mouse (#17–4010-82; 1:100) secondary antibodies, both obtained from Thermo Fisher Scientific. For immunocytochemistry, secondary antibody Alexa Fluor^TM^488 or 555-conjugated goat anti-mouse IgG_1_ (#A21121, #A21127; 1:500), IgG2a (#A21137; 1:500), IgG2b (#A21141, #21147; 1:500) or goat anti-rat (A11006; 1:500) antibodies were all obtained from Thermo Fisher Scientific. For immunoblotting, Peroxidase AffiniPure Goat Anti-Mouse IgG (H + L) (#115–035-062; 1:3000) was purchased from Jackson ImmunoResearch Europe Ltd (Ely, UK).

### Cell culture and transfection

FEMX-I cells have been authenticated by morphology and proteomics [14, 24], and are wild-type for BRAF, PTEN and NRAS [37]. FEMX-I cells lacking CD9 or overexpressing CD9-green fluorescent protein (GFP) fusion protein and those lacking CD133 (prominin-1^–^/5, hereafter clone –/5) were generated in previous studies, where they have been fully characterised [18, 37]. FEMX-I cells expressing cytochrome C oxidase subunit 8 (COX8) were generated by transfecting FEMX-I cells with mCherry-Mito-7 plasmid (a gift from Michael Davidson (#55,102; http://n2t.net/addgene:55102; RRID:Addgene_55102; Addgene, Watertown, MA) [38] using Amaxa®Cell Line Nucleofector® Kit V (VCA-1003, Lonza Biosciences) and Amaxa Nucleofector 2B device (Amaxa, Cologne, Germany) according to manufacturer instructions. 24 h after transfection, cells were selected by introducing 400 μg/ml Geneticin™ (G418 Sulfate, #10131035, Thermo Fisher Scientific) into the culture medium and further expanded.

All cells were cultured in the RPMI Medium 1640 (#21875–034, Gibco Corp., Carlsbad, CA) supplemented with 10% fetal calf serum (#A15-151, PAA Laboratories, Pasching, Austria) and 100 U/mL penicillin and 100 μg/mL streptomycin (#15140–122, Gibco Corp.) at 37 °C in a 5% CO_2_ humidified incubator. Cells lacking CD9 or CD133 as well as those with expression CD9-GFP or COX8-mCherry were maintained in the presence of puromycin (#ant-pr-1, InvivoGen, Toulouse, France) or Geneticin™ selective antibiotic. The selection agents were removed prior to the experiments.

### Flow cytometry

Cells growing in fibronectin-coated Nunc™ T-flasks (Thermo Fisher Scientific) were harvested by trypsin/EDTA treatment for 2 min at 37 °C. After trypsin inactivation, two washing steps with PBS and centrifugation (5 min at 300 × *g*), cells were resuspended in PBS containing 1% bovine serum albumin and 100 μL-cell suspension aliquots were incubated with unconjugated or fluorochrome-conjugated primary antibodies (see above) for 30 min at 4 °C. When necessary, appropriate fluorochrome-conjugated secondary antibodies were applied for 30 min at 4 °C. After washing with PBS, 20,000 events were acquired on an LSRII flow cytometer (BD Biosciences, Franklin Lakes, NJ). Instrument settings and gating strategies were established using the appropriate isotype antibody or secondary antibody. Data were analyzed using FlowJo software (FlowJo Version 10.9.0, LLC, Ashland, OR). The median fluorescence intensity (MFI) was calculated as the difference between the MFI values obtained from the stained and negative controls (i.e. cell populations incubated with isotype primary antibody or secondary antibody alone).

### Scanning electron microscopy

FEMX-I cells were grown on coverslips coated with 0.01% poly-L-lysine for 1–2 days. They were fixed in 2% glutaraldehyde (#00216–30, Polysciences Inc., Warrington, PA) for 1 h at room temperature and then overnight at 4 °C. Following 2-h post-fixation in 1% osmium tetroxide (#E19190, Electron Microscopy Sciences, Hatfield, PA) at 4 °C, they were subjected to dehydration in an acetone gradient (25–100%) and critical point-dried in a CO_2_ system (Critical Point Dryer, Leica Microsystems, EM CPD 300, Wetzlar, Germany). Afterward, samples were sputter-coated with gold or platinum (sputter-coating device SCD 050; BAL-TEC GmbH, Witten, Germany) and examined at a 5-kV or 15-kV accelerating voltage in a field emission-scanning electron microscope (Jeol JSM 7500F, Freising, Germany) or at a 15-kV accelerating voltage using tabletop microscope (HITACHI TM1000, Krefeld, Germany) with solid-state backscattered electron detector.

### Transmission electron microscopy

FEMX-I cells were grown for 1–2 days in 35-mm Petri dishes. Cells were then pre-fixed by addition of an equal volume of 4% paraformaldehyde (PFA) into the cell culture medium. After one hour, cells were fixed in 4% PFA, 0.1 M CaCl_2_ with or without 1% glutaraldehyde in phosphate buffer overnight at room temperature or at 4 °C. After washing, cells were incubated in 1% aqueous osmium tetroxide for one hour at room temperature. Cells were then washed with water and contrasted with 0.5% aqueous uranyl acetate for 30 min at room temperature. Afterward, samples were processed through a graded series of ethanol for standard plastic embedding in LX 112 resin (LADD Research, VT). Sheets of resin embedded cells were removed from the Petri dishes by liquid nitrogen. Sections were cut at 70-nm on an UCT ultramicrotome (Leica Microsystems) parallel to the cell support (Petri dish), and post-stained with uranyl acetate and lead citrate. The samples were viewed in a Morgagni or Tecnai Biotwin T12 electron microscope (Thermo Fisher Scientific, former FEI, former Philips) and images acquired on a Morada CCD camera (EMSIS Münster Germany, former Olympus, former SIS) or F416 CMOS camera (TVIPS, Gilching, Germany).

### Fluorescence-labeling and confocal laser-scanning microscopy

FEMX-I cells were cultured on poly-L-lysine- or fibronectin-coated coverslips or ibidi μ-slides (#80826, ibidi GmbH, Gräfelfing, Germany) or glass-bottom 35-mm dishes (#P35G-1.5–14-C, MatTek Corp., Ashland, MA) for 1 or 2 days. After washing with PBS, cells were fixed in 4% PFA in PBS for 30 min at room temperature and then incubated for 10 min in PBS containing 50 mM NH_4_Cl. Cells were then blocked in 0.2% gelatin/PBS for 30 min at room temperature. For the intracellular detection of antigens, cells were blocked and permeabilized with 0.2% saponin (#A2542.0100, AppliChem GmbH, Darmstadt, Germany) diluted in 0.2% gelatin/PBS. Afterward, cells were labeled with primary antibodies diluted in blocking/permeabilization buffer for 30 min followed by the appropriate secondary antibody conjugated to Alexa Fluor®488 or 555 for 30 min at room temperature. For the visualization of lipid droplets, Bodipy®493/503 (1:500) or LipidSpot™ 488 or 610 (1:1000) dyes were added in the last incubation step. To detect mitochondria, MitoTracker Red CMXRos (100 nM) or MitoView™ Fix 640 (100 nM) were added to cell culture medium for 2 h, before cell fixation. To highlight the cell membrane, cells were labeled with fluorochrome-conjugated WGA (1:400) by incubating in the PBS containing 1 mM CaCl_2_ and 0.5 mM MgCl_2_. Nuclei were visualized with 4′,6-diamidino-2-phenylindole (DAPI, 1 μg/ml). Finally, cells were washed thrice in PBS, twice in H_2_O and mounted in Mowiol®4–88 (#475904, Calbiochem/Merck KGaA, Darmstadt, Germany) prior observation with Leica SP5 upright confocal laser-scanning microscope (Wetzlar, Germany). In some experiments, cells were not mounted but were directly observed with Zeiss LSM 780 inverted confocal laser-scanning microscope (Carl Zeiss AG, Jena, Germany). The images acquired under the same setting for all cell lines were processed with Fiji software [39] and figures were prepared with Adobe Illustrator (Adobe Inc., CA).

### Time-lapse video microscopy

For live cell imaging, cells growing on fibronectin-coated glass-bottom dishes for 24 h were incubated either with LipidSpot™ 610 or BODIPY™ 493/503 Lipid Droplet Stains for 2 h, prior time-lapse video microscopy. Imaging was performed with widefield fluorescence microscopes (Zeiss Axiovert 200 M with 20x/0.8 Ph2 objective or Zeiss Axio Observer.Z1, inverted with 20x/0.8 Ph2 Plan-Apochromat Air objective, Jena, Germany). The microscopes were equipped with an incubation chamber allowing imaging at 37 °C under 5% CO_2_ atmosphere. Images were taken at 2–5 min intervals over a period of 12 h. All images were processed with Fiji.

### Differential centrifugation and cell solubilization

FEMX-I cells were grown in Costar® six-well plates (#3516, Corning Inc.) for 1 or 2 days, washed with PBS and supplied with fresh medium (see above, 2 ml) for 24 h. Afterward, conditioned media were collected, supplemented with Complete™ protease inhibitor cocktail (#11836145001; Roche Diagnostics GmbH, Mannheim, Germany) and then subjected to differential centrifugation as follows: 5 min at 400 × *g*; supernatant, 20 min at 1,200 × *g*; supernatant, 30 min at 10,000 × *g*; supernatant, 1 h at 200,000 × *g* [40]. For the ultracentrifugation, we used a LA-110 Fixed-Angle Rotor (Beckman Coulter, Krefeld, Germany). All steps were performed at 4 °C. Proteins in the 200,000* g* supernatant were concentrated by methanol/chloroform (4:1) precipitation [41]. The resulting pellets were resuspended in Laemmli sample buffer and analyzed by immunoblotting. In parallel, FEMX-I cells were harvested and centrifuged for 5 min at 300 × *g*. Cell pellets were lysed in solubilization buffer (1% NP-40, 0.5% sodium deoxycholate, 0.1% sodium dodecyl sulfate (SDS), 150 mM sodium chloride, 50 mM Tris/HCl, pH 7.5) supplemented with the protease inhibitor cocktail (see above) for 30 min at 4 °C. Detergent extracts were centrifuged (10 min, 16,000 × *g*) at 4 °C, and the resulting supernatants were mixed with Laemmli sample buffer (4X).

### Immunoblotting

Solubilized proteins were separated by SDS–polyacrylamide-gel electrophoresis (7.5%) and transferred to a polyvinylidene difluoride membrane (pore size 0.45 µm; Millipore Corp, Bedford, MA) using a semi-dry transfer system (Cti, Idstein, Germany). After transfer, membranes were incubated in blocking buffer (PBS containing 5% low fat milk powder and 0.3% Tween®20) overnight at 4 °C prior to being probed with either monoclonal antibody 80B258 or AP125 directed against CD133 and adipophilin/perilipin 2, respectively, for 1 h at room temperature. Antigen–antibody complexes were detected using appropriate horseradish peroxidase (HRP)-conjugated secondary antibody and visualized with enhanced chemiluminescence reagents (ECL system; GE Healthcare Life Sciences). Membranes were exposed to Amersham Hyperfilms ECL (GE Healthcare Life Sciences) and developed using Optimax Mammo X-Ray film processor (Protec, Oberstenfeld, Germany).

### Statistical analysis and reproductivity

Statistical analyses were performed using GraphPad software (Version 9.4.1, Boston, MA). For analysis of cellular processes and extracellular particles containing mitochondria after CCCP treatment, a Mann–Whitney U test was used. Cell extremity analysis was carried out by analyzing five random cell fields, each with a surface area of 0.056 mm^2^, from two independent experiments. Analysis of mitochondria-containing extracellular lipidosomes was performed by selecting 35 particles based on WGA staining in each experiment (*n* = 3) and screening for the presence or absence of lipid droplets and/or mitochondria. All data are shown as the mean ± standard deviation (S.D.). All immunocytochemical staining, time-lapse video microscopy, differential centrifugation, immunoblotting and sample preparations for scanning electron microscopy (SEM) were carried out in at least three independent experiments, and representative data are shown. Sample preparation for transmission electron microscopy (TEM) was carried out twice.

## Results

Numerous types of EV have been described in the literature [42], including those with small diameter (< 150 nm) such as exosomes and ectosomes that are released when multivesicular bodies fuse with the plasma membrane or by direct budding from plasma membrane, notably protrusions, respectively [43, 44]. Their release by melanoma cells was previously documented [18]. Here, we investigated whether other types of EVs could be generated by melanoma cells, impacting the melanoma microenvironment.

**Fig. 1:**
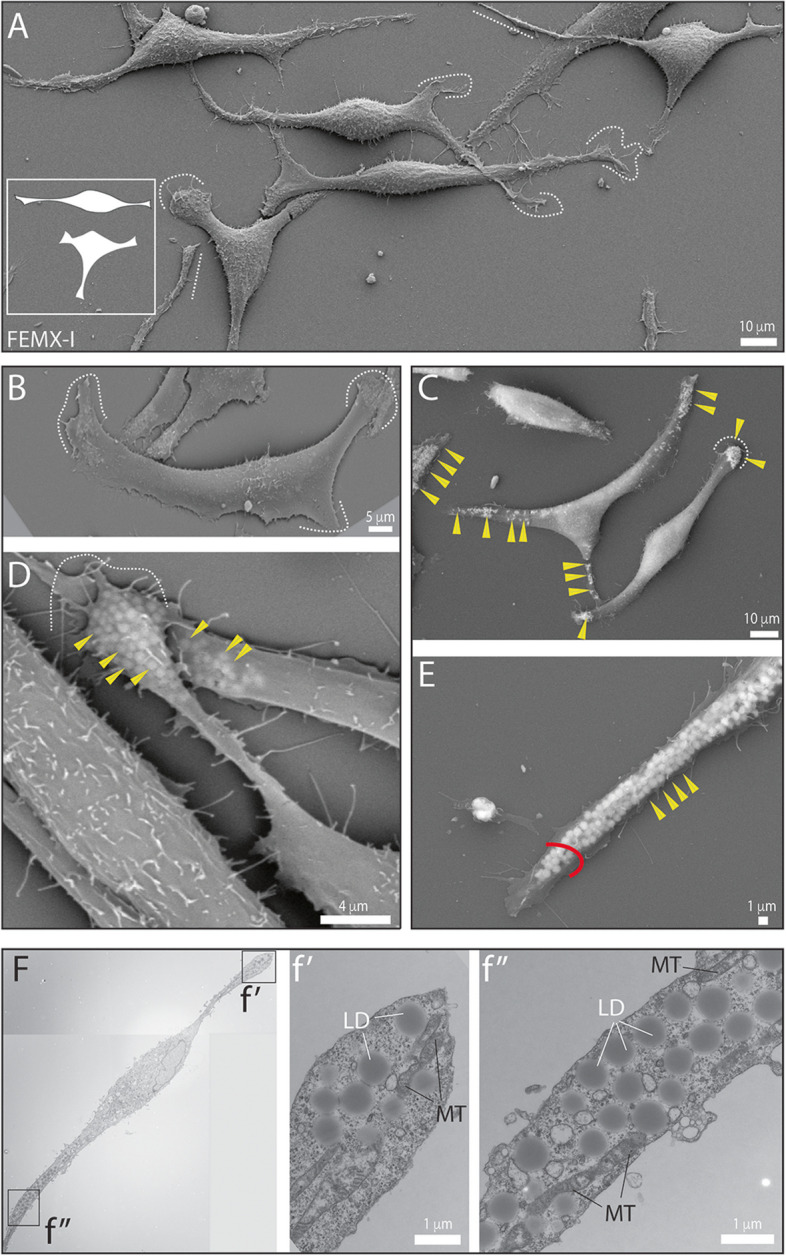
Melanoma FEMX-I cells harbor bipolar or triangular morphotypes and contain lipid droplets and mitochondria at their extremities. **A**-**F** FEMX-I cells growing on poly-L-lysine-coated coverslips (**A**-**E**) or on plastic Petri dishes (**F**) were processed for scanning (**A**-**E**) and transmission (**F**) electron microscopy. Two main morphotypes are observed; cells with a bipolar and tripolar shape (see illustration in **A**). Their extremities are either short and flattened with filopodia adhering to the support (**A**-**D**, dashed line) or long and narrow (**A**, **E**, straight dotted line and red arc). Various small, rounded particles are observable particularly at the extremities of the cells when applying a higher acceleration voltage (15 kV instead 5 kV) during SEM analysis (**C**-**E**, yellow arrowhead). These homogeneous intracellular particles appear as opaque spheres by TEM, which is the typical appearance of lipid droplets (LD, **F**, see enlargement of images f’ and f’’). Mitochondria (MT) are present in the vicinity of lipid droplets (see also Additional file 1: Fig. S2). Scale bars are indicated

For this purpose, we used FEMX-I cells. Upon culture on poly-L-lysine- or fibronectin-coated surfaces, they appeared with a bipolar or triangular morphology and their extremities were of different lengths, as observed at high resolution by SEM (Fig. 1A). Such morphologies were in line with our previous studies [18, 45]. The cell endpoints were either widespread with emerging short filopodia (Fig. 1A, B, dashed curved line) or very narrow, especially for the long extensions (Fig. 1A, straight dashed line). The interaction between the tetraspanin membrane protein CD9 (also called Motility-related protein 1) and the integrin β1 could explain the spreading of cell extremities [46, 47] (reviewed in Ref. [48]). Indeed, the analysis of FEMX-I cells lacking CD9 or overexpressing CD9-GFP fusion protein [37] revealed that narrow cell extremities were observed after depletion of CD9, but not after its overexpression (Additional file 1: Fig. S1A-C).

Interestingly, when we increased the interaction volume by higher accelerating voltage in our SEM analysis, we observed various small rounded particles that were concentrated at the cell extremities (Fig. 1C, D). In straight and narrow extensions, such particles were often aligned (Fig. 1E). The absence of CD9 did not prevent this phenomenon, whereas overexpression of CD9-GFP gave an identical pattern to that of the wild-type cells (Additional file 1: Fig. S1D-H). To gain a deeper understanding of these cytoplasmic spherical structures, we carried out TEM analysis. They appeared as homogeneous, opaque spheres with a monolayer surrounding them, typical of lipid droplets (Fig. 1F). Mitochondria were also detected at cell extremities and they were often in contact with the presumed lipid droplets (Fig. 1F and Additional file 1: Fig. S2A, B).

**Fig. 2:**
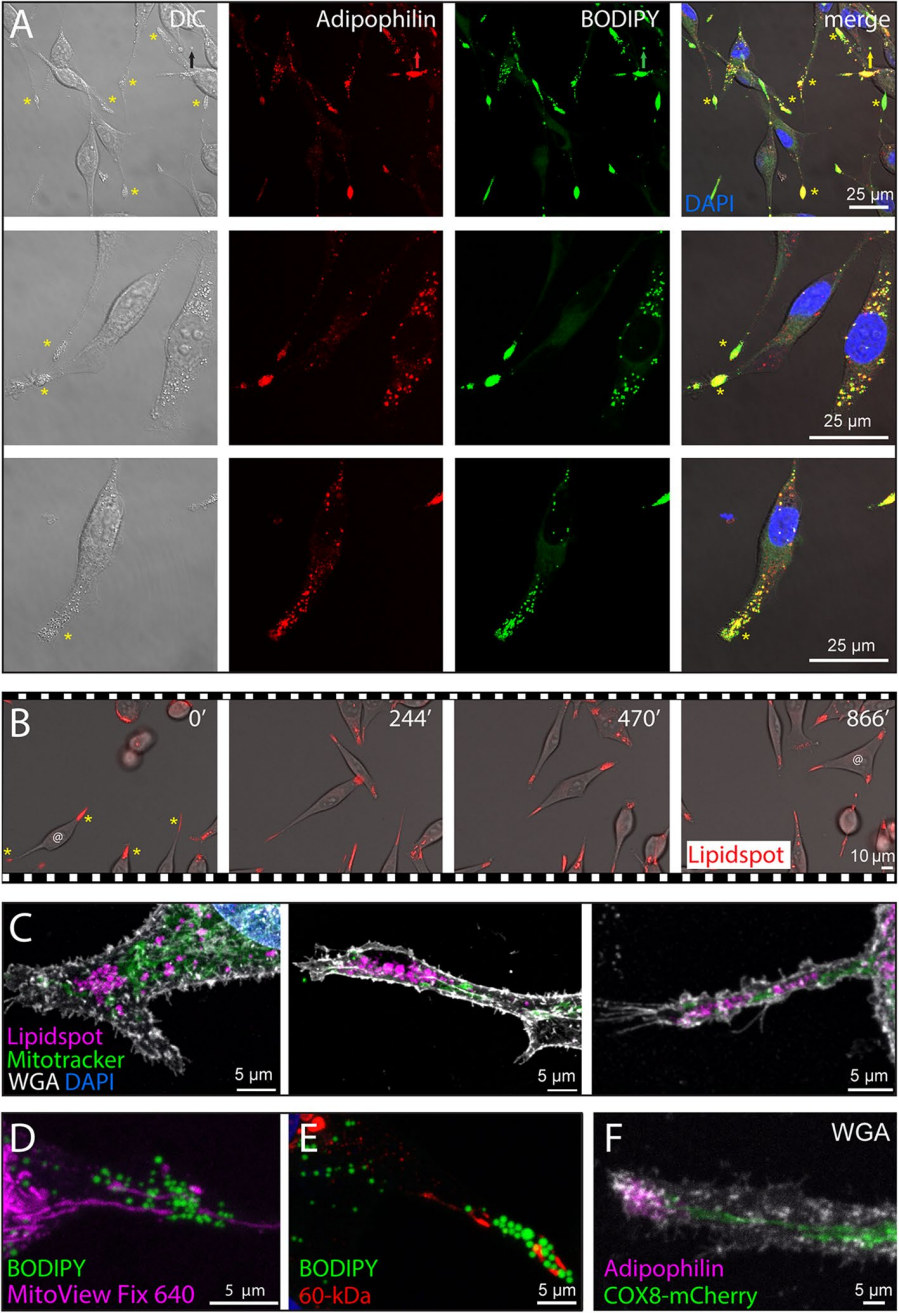
Fluorescence detection of lipid droplets and mitochondria at the extremities of melanoma cells. **A**-**F** Native FEMX-I cells (**A**-**E**) or COX-8-mCherry-transfected cells (**F**) growing either on poly-L-lysine (**A**) or fibronectin-coated (**B**-**F**) supports were processed for CLSM (**A**, **C**-**F**) or live-cell phase-contrast/fluorescence video microscopy (**B**). PFA-fixed and saponin-permeabilized cells were immunolabeled with an anti-adipophilin (**A**, **F**) or anti-60-kDa mitochondrial antigen (**E**) antibody and/or stained with fluorescent dyes BODIPY™ 493/503 (**A**, **D**, **E**) or LipidSpot™ 610 (**B**, **C**). Alternatively, cells were co-stained with MitoTracker™ Red CMXRos (**C**) or MitoView™ Fix 640 (**D**). Cells were counterstained with DAPI (**A**, **C**, blue) or fluorescence-conjugated WGA (**C**, **F**) to highlight nuclei and glycoconjugates at the cell membrane, respectively. LipidSpot-stained cells were observed alive and elapsed time in minutes is shown on the top-right corner (**B**). Samples were pseudo-colored with a given marker as indicated. The images presented in panel **B** are excerpted from the Additional file 2: Video S1. Asterisks indicate the enrichment of lipid droplets at the cell extremities (**A**, **B**), while the symbol @ shows a cell changing from a bipolar to a tripolar morphology over time (**B**). The arrow points to a small EV containing lipid droplets (**A**). DIC, differential interference contrast. Scale bars are indicated

To confirm that these spherical structures were indeed lipid droplets, we labeled FEMX-I cells with antibodies specific for adipophilin/perilipin-2, a lipid droplet-associated protein [49, 50], and/or stained them with either BODIPY™ 493/503, which labels neutral lipids, or LipidSpot™ 488/610 stain, a fluorogenic neutral lipid dye that rapidly accumulates in lipid droplets. After PFA-fixation and cell permeabilization, adipophilin and BODIPY™ 493/503 labeled the same structures as observed by confocal laser-scanning microscopy (CLSM). Consistent with SEM observations, they were highly concentrated at the cell extremities (Fig. 2A). Live cell-imaging of LipidSpot-labeled FEMX-I cells confirmed these data (Fig. 2B and Additional file 2: Video S1). Time-lapse video microscopy showed that these cells are highly dynamic, as they can change from a bipolar to a tripolar form. Interestingly, the formation of the third extremity involved a redistribution of lipid droplets, either during the cleavage of the 'mother' extremity or afterwards, with a translocation of lipid droplets from one “daughter” extremity to the other (Additional file 1: Fig. S3A, B, Additional file 2: Video S1 and Additional file 3: Video S2). The mechanism underlying this symmetrical/asymmetrical distribution of lipid droplets will need further investigation (Additional file 1: Fig. S3C). Besides these lipidic structures, the staining of FEMX-I cells with either MitoTracker™ Red CMXRos or MitoView™ Fix 640, or the immunolabeling for a 60-kDa mitochondrial antigen confirmed the presence of mitochondria in their extremities, in agreement with TEM images (Fig. 2C-E). Transfection of cells with the mitochondrial matrix marker COX8-mCherry also showed the presence of mitochondria in cell extremities (Fig. 2F). Like lipid droplets, mitochondria often appeared aligned, regardless of the labeling methods, suggesting their collective movement along these extremities. Overall, these data revealed that melanoma FEMX-I cells accumulated lipid droplets and mitochondria in their cell extremities.

### Identification of a novel structure referred to as extracellular lipidosomes

**Fig. 3:**
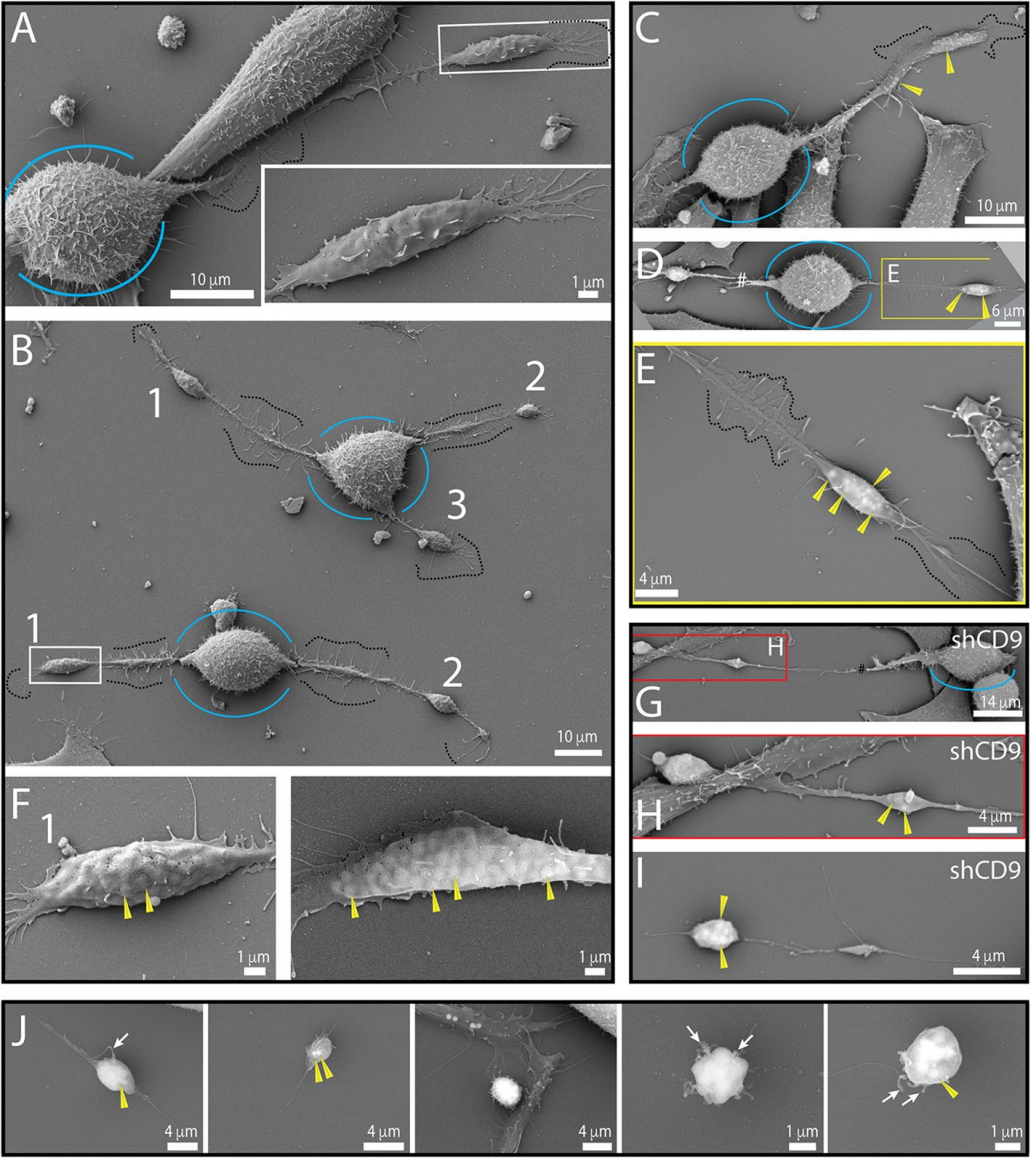
Cell extremities during cell division and extracellular lipidosomes. **A**-**J** FEMX-I (**A**-**F**, **J**) or CD9-deficient FEMX-I (shCD9, **G**-**I**) cells growing on poly-L-lysine-coated glass coverslips were processed for SEM using 5-kV (**A**, **B** and **F**, left panel) or 15-kV (**C**-**E**, **F**, right panel, **G**-**J**) accelerating voltage. Note the presence of large lipid droplet-filled membrane structures (e.g., numbered 1–3 in **B**) connected with a membrane bridge containing numerous filopodia (**A**-**C**, **E**, dashed lines) to rounded cell body during cell division (**A**-**H**, blue line). Filopodia are barely present in shCD9 cells (**G**-**I**). Cell extremities can detach completely from the cells, resulting in the formation of larger EVs containing various lipid droplets (**I**, **J**). Some regions of interest indicated by colored boxes (**A**, **B**, **D**, **G**) have been enlarged (**A**, **E**, **F**, **H**) as indicated. The yellow arrowhead points to a lipid droplet, while the white arrow points to a microvillar-like structure on extracellular lipidosomes. The symbol # indicates a membrane rupture during sample preparation. Scale bars are indicated

In addition to the elongated and polar morphologies of FEMX-I cells, SEM analysis also revealed several rounded cells suggesting cell division (Fig. 3A, B). During the cell rounding process at mitotic entry [51, 52], cellular extremities retracted and numerous filopodia appeared throughout the remaining long cell process and, interestingly, a residual blob-like structure appeared at or near the extremity (Fig. 3A-E). Again, by applying a higher acceleration voltage, we could observe the presence of lipid droplets in these structures (Fig. 3C-F). They were also present in CD9-deficient cells, but small filopodia along cellular processes were rare (Fig. 3G-H). Unexpectedly, these blob-like particles could be found completely detached from the originating cells (Fig. 3I, J). Their size varied from 2 to 6 µm and remnants of the cellular process were occasionally associated with them at the substrate level. Other tiny membrane processes, such as microvillus-like structures, were present on their surface. Around 90% of these free blob-like particles contained numerous lipid droplets, which often seemed to be highly compacted (Fig. 3J; for quantification, see below). As they contained lipid droplets and are released into the extracellular space, we will refer to them from now on as “*extracellular lipidosomes*”.

### Biogenesis of extracellular lipidosomes and their uptake by other cells

**Fig. 4:**
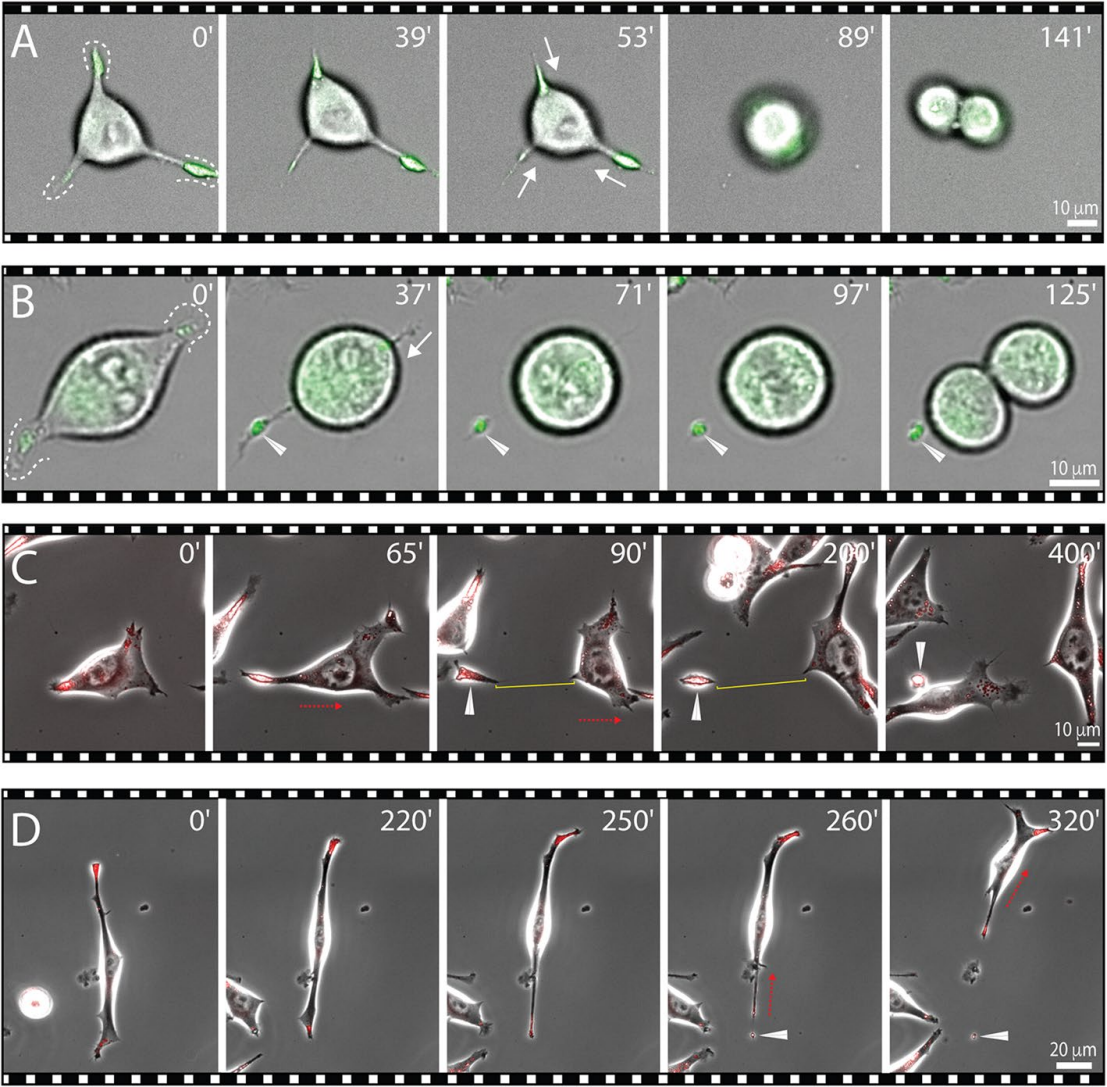
Biogenesis of extracellular lipidosomes occurs during cell division or cell migration. **A**-**D** FEMX-I cells growing on fibronectin-coated supports were recorded in live by phase-contrast/fluorescence video microscopy after staining with LipidSpot™ 488/610. During cell division, cells can reabsorb material from their extremities (**A**, dashed line and white arrow) or lose it, resulting in the formation of extracellular lipidosomes (**B**, white arrowhead). During migration, cells can release extracellular lipidosomes (**C**, **D**, white arrowhead). Extracellular lipidosomes can be either large (**B**, **C**) or small (**D**). The very thin process linking an extracellular lipidosome to the donor cell can withstand traction before breaking (**C**, yellow bracket). Red arrow indicates the orientation of cell migration. Elapsed time in minutes is shown on the top-right corner. The images are excerpted from the Additional file 4: Video S3 (**A**, top), Additional file 6: Video S5 (**B**), Additional file 7: Video S6 (**C**) and Additional file 8: Video S7 (**D**). Scale bars are indicated

To unravel the biogenesis of extracellular lipidosomes, we performed live imaging of dividing FEMX-I cells after their incubation with LipidSpot™ 488/610 dyes. We observed that, during their division, cells reabsorbed their long processes, including their extremities containing lipid droplets (Fig. 4A, Additional file 4: Video S3 and Additional file 5: Video S4). In other cases, it appeared that one of the long processes did not fully retract, resulting in the formation of extracellular lipidosomes (Fig. 4B and Additional file 6: Video S5). In rare cases, similar larger (> 2 µm) or smaller (< 2 µm) lipid droplet-containing structures were also generated during the process of cell migration and left behind a motile cell (Fig. 4C, D, Additional file 7: Video S6 and Additional file 8: Video S7; see also Fig. 2A, arrow). In these instances, the long, thin processes connecting the future extracellular lipidosome to the cell remained stable for some time before rupture (Fig. 4C, bracket). Unlike with other cell types, we did not observe the formation of retraction fibers behind the migrating cells, nor the formation of migrasomes (data not shown, see “Discussion” section). Thus, the biogenesis of extracellular lipidosomes is mainly based on cell division in which a complete retraction of the cellular process does not occur.

**Fig. 5:**
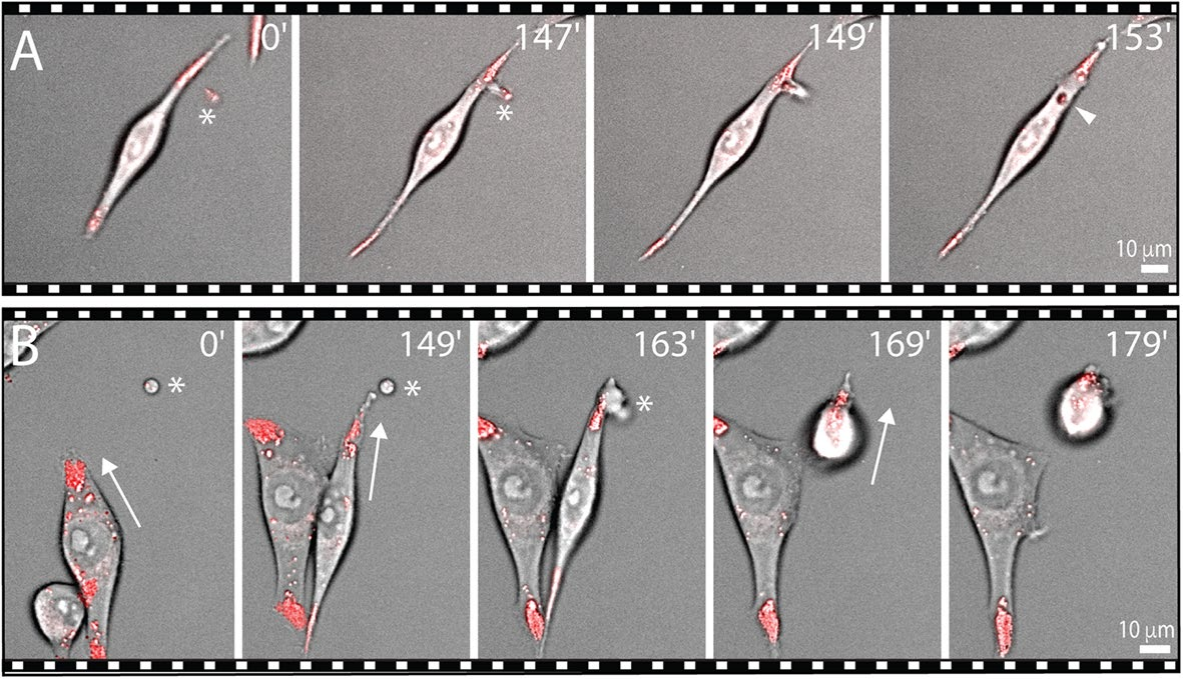
Extracellular lipidosomes are taken up by non-migrating and migrating cells. **A**, **B** FEMX-I cells growing on fibronectin-coated supports were recorded in live by phase-contrast/fluorescence video microscopy after staining with LipidSpot™ 610. Non-migrating (**A**) and migrating (**B**, arrow) cells can uptake an extracellular lipidosome (asterisk). The latter can be detected inside the recipient cells (**A**, arrowhead). Elapsed time in minutes is shown on the top-right corner. The images are excerpted from the Additional file 9: Video S8 (**A**) and Additional file 10: Video S9 (**B**). Scale bars are indicated

Next, we monitored by live imaging of LipidSpot-stained cells the fate of extracellular lipidosomes after their release. Interestingly, FEMX-I cells, whether migrating or not, could dynamically take up extracellular lipidosomes (Fig. 5A and B, respectively, Additional file 9: Video S8 and Additional file 10: Video S9). Moreover, their uptake seemed somehow directed, which suggests that a chemoattractant might play a role in this process (see “Discussion” section).

### Characterization of the cytoskeleton at the extremities of melanoma cells and the extracellular lipidosomes derived therefrom

**Fig. 6:**
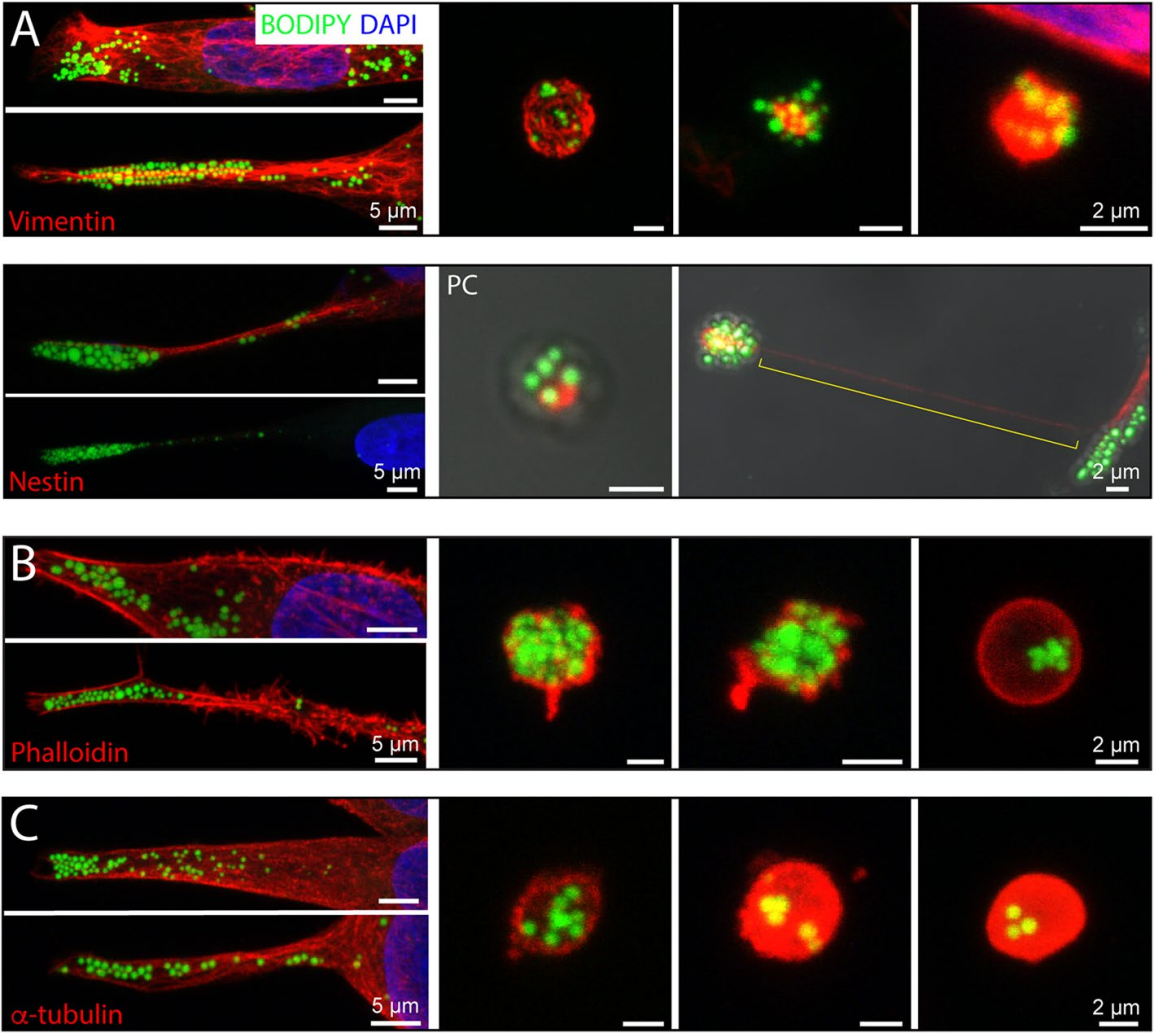
Cytoskeleton components associated with extracellular lipidosomes. **A**-**C** FEMX-I cells growing on fibronectin-coated supports were processed for CLSM. PFA-fixed and saponin-permeabilized cells were immunolabeled with anti-Vimentin, Nestin (**A**) or α-tubulin (**C**) antibodies or stained with fluorochrome-conjugated phalloidin (**B**). All samples were co-stained with the fluorescent dye BODIPY™ 493/503 and counterstained with DAPI to highlight lipid droplets and nuclei, respectively. Composite images of all x–y optical sections are shown. Cell extremities and extracellular lipidosomes are displayed in the left and right panels, respectively. The yellow bracket indicates the very thin Nestin^+ ^process linking an extracellular lipidosome to the donor cell prior rupture (**A**). PC, phase contrast image. Scale bars are indicated

The morphological organization of cells is highly dependent on cytoskeletal components such as intermediate filaments, actin filaments and microtubules. This prompted us to examine their potential presence at the cell extremities and in the extracellular lipidosomes derived therefrom. We immunolabeled FEMX-I cells for intermediate filaments Vimentin and Nestin. Cells were also stained with BODIPY™ 493/503 to highlight lipid droplets prior to CLSM analysis. Both types of intermediate filaments were observed in the structures of interest; cell extremities and extracellular lipidosomes (Fig. 6A, left and right panels, respectively). Note that not all cells were positive for Nestin (about 30% negative), suggesting that it is not required for the accumulation of lipid droplets in cell extremities (Fig. 6A, lower left panel). In contrast, cytokeratins were absent from these non-epithelial cells, as revealed by the use of a Pan-cytokeratin antibody (data not shown). The presence of intermediate filaments in the cell extremities was confirmed by TEM analysis, where they appeared in close proximity to lipid droplets (Additional file 1: Fig. S2C, D). Then, cells were either stained with fluorochrome-conjugated phalloidin or immunolabeled for α-tubulin antibody together with BODIPY™ 493/503 staining. Actin filaments and microtubules were, like intermediate filaments, detected in cell extremities and extracellular lipidosomes (Fig. 6B and C, respectively). Of these four cytoskeletal components, only Nestin was found in a very thin process connecting an extracellular lipidosome to the donor cell, as shown above when these large EVs were produced by a mechanism based on cell migration (Fig. 6A, lower right panel, bracket). Thus, we found different types of cytoskeletal components in these structures, which could contribute (i) to the targeting and/or stability of lipid droplets at cell extremities; (ii) to their redistribution after cleavage of such an extremity; and/or (iii) to the formation of extracellular lipidosomes during cell division and migration.

### Characterization of integrins at the extremities of melanoma cells and in the extracellular lipidosomes

**Fig. 7:**
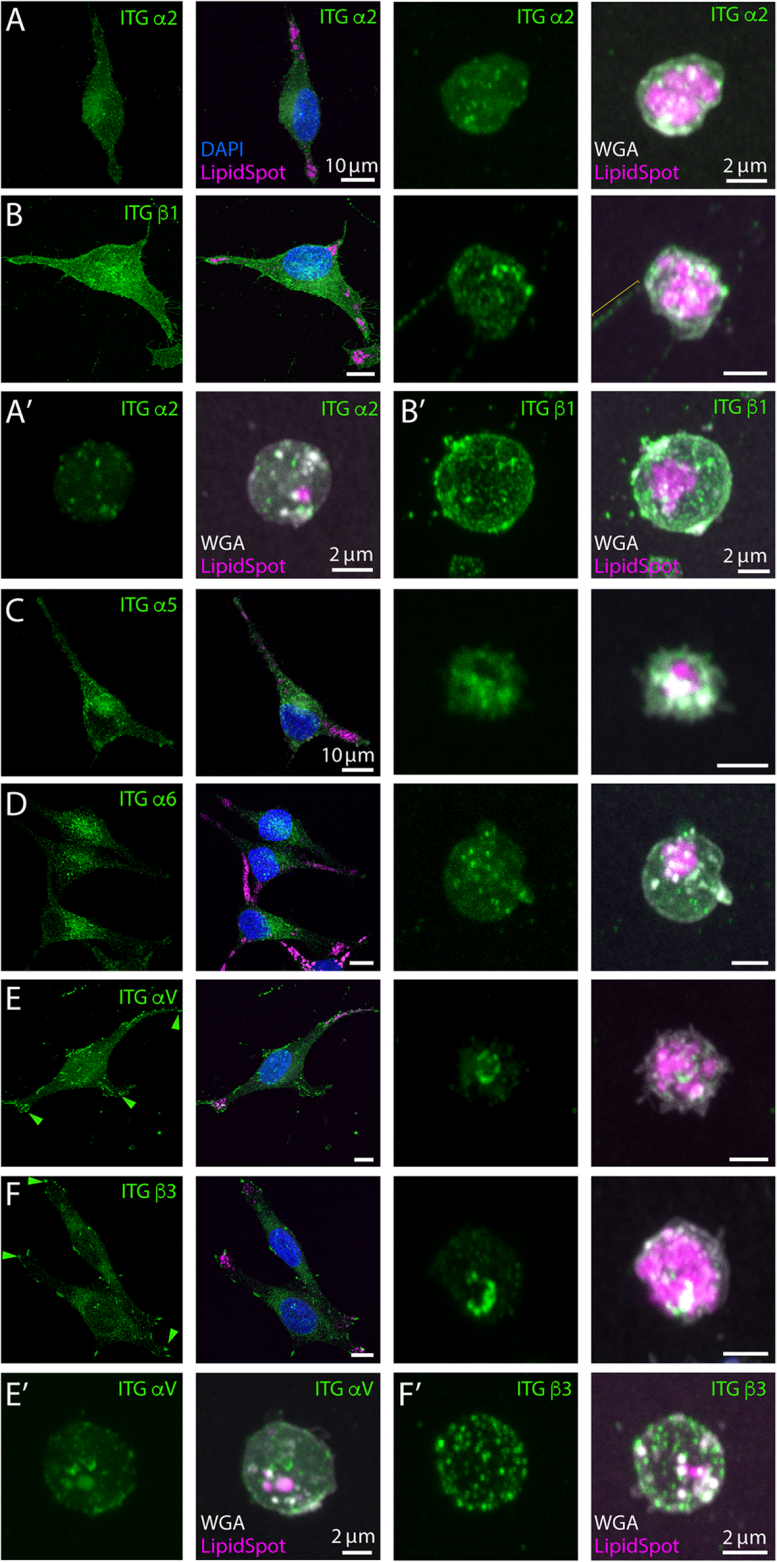
Expression of integrins at cell extremities and extracellular lipidosomes. **A**-**F** FEMX-I cells growing on fibronectin-coated supports were processed for CLSM. PFA-fixed and saponin-permeabilized cells were immunolabeled with various anti-integrin (ITG) antibodies as indicated and co-stained with fluorescent dyes LipidSpot™ 610 and fluorescence-conjugated WGA to highlight lipid droplets and glycoconjugates, respectively. Samples were counterstained with DAPI to highlight nuclei. Composite images of all x–y optical sections are shown and a given marker was pseudo-colored as indicated. Cells and extracellular lipidosomes are shown in the left and right panels, respectively, except for those in panel **A**’-**F**’. The yellow bracket indicates the very thin ITG β1^+^ process linking an extracellular lipidosome to the donor cell prior rupture, while arrowhead indicates specific ITG at cell extremities. Scale bars are indicated

Given the attachment to substrates of cell extremities and extracellular lipidosomes, we investigated whether certain ITG proteins were present. Flow cytometry analysis of trypsinized and immunolabeled cells revealed expression of ITG α2, α5, α6, αV and β1 and β3 in FEMX-I cells, while αM, αX, αE (AE), α2b, α4, β2 and β4 were absent or below detection level (Table 1). This information prompted us to examine their potential association either with cell extremities, extracellular lipidosomes or both. To this end, cells growing on fibronectin-coated supports were immunolabeled for a given ITG and then co-stained with LipidSpot™ 610 and fluorescence-conjugated WGA to highlight lipid droplets and glycoconjugates, respectively, prior to CLSM analysis. Although all expressed ITGs were present both in cells and in the extracellular lipidosomes derived from them, their quantity and/or distribution varied (Fig. 7). For instance, ITG α2 and β1 were evenly distributed in the cells between cell body and extremities (Fig. 7A, B), whereas ITG α5 and α6 were less abundant at the cell extremities (Fig. 7C, D). In contrast, both ITG αV and β3 occurred in small clusters similar to focal adhesions, which were sparsely distributed with an enrichment at the cell extremities (Fig. 7E, F; Table 1). ITG αVβ3 acts as a receptor for Vitronectin, promoting cell adhesion and spreading, which contributes to melanoma metastasis [53–55]. While ITG α2, α5, α6 and β1 were distributed equally on extracellular lipidosomes with various levels of expression (Fig. 7A-D, A’, B’), ITG αV and β3 were often concentrated in limited areas, in close contact with the substrate (Fig. 7E, F), reflecting their concentration in focal adhesions [56]. However, they were also occasionally observed on whole extracellular lipidosomes (Fig. 7E’, F’).

**Table 1:** Integrin expression in FEMX-I cells

Integrin	Cluster of differentiation	Expression as detected by flow cytometry^a^	Accumulation at cell extremities as detected by ICH
alpha M	CD11b	–	N.D.
alpha X	CD11c	–	N.D.
alpha 2b	CD41a	–	N.D.
alpha 2	CD49b	+ +	No
alpha 4	CD49d	–	N.D.
alpha 5	CD49e	+	No
alpha 6	CD49f	+	No
alpha V	CD51	+ + +	Yes
alpha E (AE)	CD103	–	N.D.
beta 1	CD29	+ + +	No
beta 2	CD18	–	N.D.
beta 3	CD61	+ +	Yes
beta 4	CD104	–	N.D.
alpha V/beta 3	CD51/61	+ + +	Yes^b^

*MFI* Median fluorescence intensity (arbitrary unit), *ICH* Immunocytochemistry, *N.D.* Not determined

^a^Cell surface expression is defined as: –, MFI < 400; + , 400 ≤ MFI < 1000; +  + , 1000 ≤ MFI < 10,000; +  +  + , MFI ≥ 10,000

^b^Data not shown

### Occurrence of mitochondria in extracellular lipidosomes

**Fig. 8:**
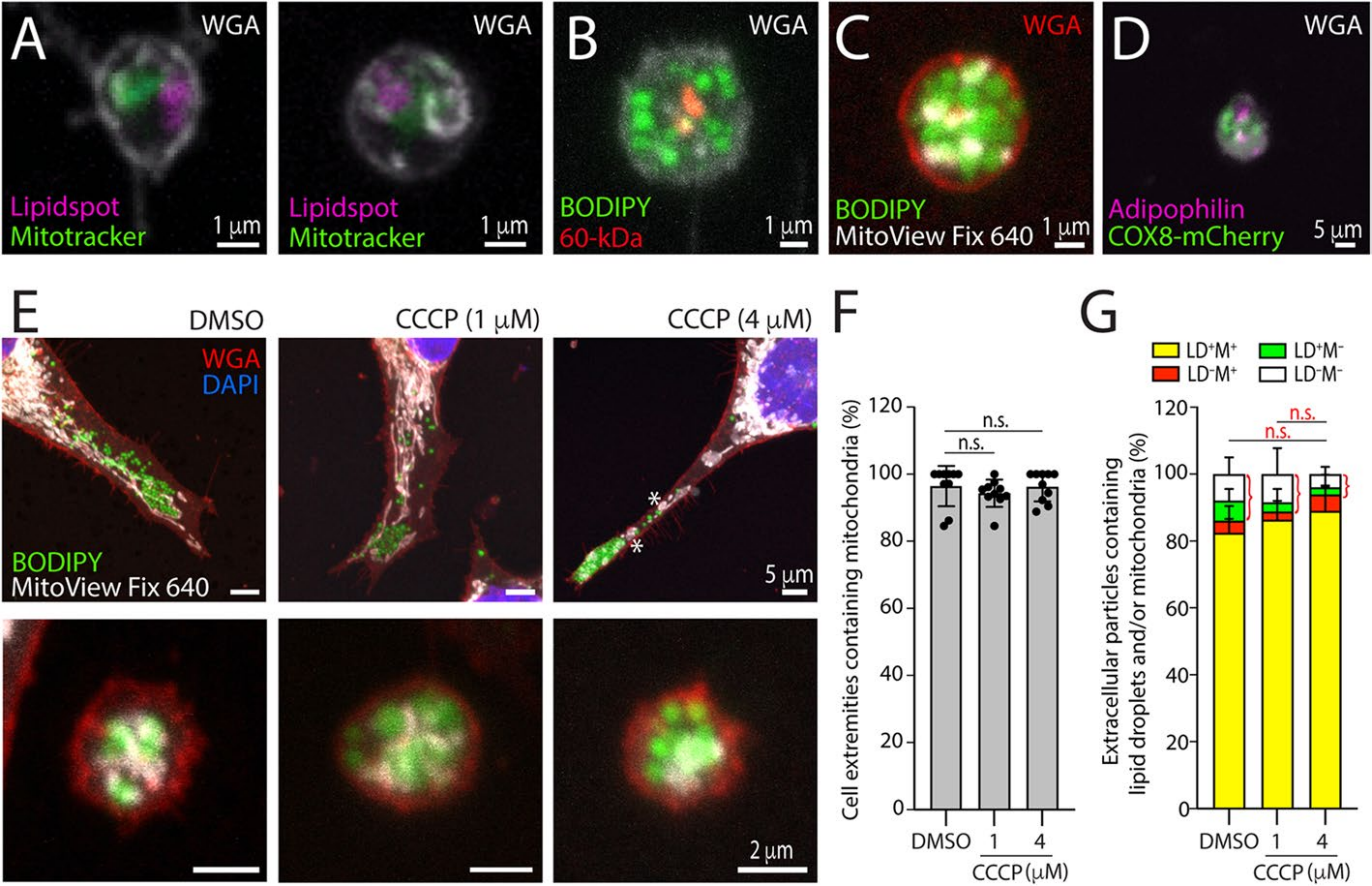
Extracellular lipidosomes contain mitochondria. **A**-**D** Native (**A**-**C**) or COX-8-mCherry-transfected (**D**) FEMX-I cells growing on fibronectin-coated supports were processed for CLSM. Cells preincubated with MitoTracker™ Red CMXRos (**A**) or MitoView™ Fix 640 (**C**) were PFA-fixed and co-stained with fluorescent dyes LipidSpot™ 610 (**A**) or BODIPY™ 493/503 (**C**) or saponin-permeabilized, immunolabeled with an anti-60-kDa mitochondrial antigen antibody and stained with BODIPY™ 493/503 (**B**). Alternatively, COX-8-mCherry-transfected cells were permeabilized and immunolabeled with an anti-adipophilin antibody (**D**). Samples were also co-stained with fluorescence-conjugated WGA to highlight glycoconjugates at the cell membrane. **E**–**G** Native FEMX-I cells were incubated without (DMSO, control) or with 1 or 4 µM of CCCP for 8 h and for the 2 last hours were additionally co-incubated with MitoView™ Fix 640. After fixation and staining with BODIPY™ 493/503, cells were co-stained with fluorescence-conjugated WGA and DAPI to highlight and glycoconjugates at the cell membrane and nuclei, respectively. In all cases, composite images of all x–y optical sections are shown and a given marker was pseudo-colored as indicated. Cell extremities (upper panels) and the extracellular lipidosomes (lower panels) are shown (**E**). Note that in drug-treated cells, mitochondria are rounded (asterisk). Mitochondria-containing and mitochondria-free cell extremities (**F**) and extracellular particles (**G**) were quantified under various conditions as indicated. More than 200 cell extremities and 100 extracellular particles per condition were analyzed from 2 and 3 independent experiments, respectively. Each individual data in panel **F** corresponds to the percentage of cell extremities found in an area of 0.056 mm^2^. Extracellular particles were classified as containing or not lipid droplets (LD) and mitochondria (M), as indicated (**G**). Mean ± S.D. are presented. Although not significant, a slight reduction in extracellular particles without mitochondria (green and white, red bracket) is observed after incubation with CCCP. N.s. not significant, Scale bars are indicated

As described above, mitochondria were found in the proximity of lipid droplets at the cell extremities. Since this cellular subdomain is the donor membrane for extracellular lipidosomes, we determined whether the latter also contained mitochondria. To this purpose, FEMX-I cells were stained with different combinations of fluorescent dyes marking either lipid droplets (LipidSpot™ 610, BODIPY™ 493/503) or mitochondria (MitoTracker™ Red CMXRos, MitoView™ Fix 640), or immunolabeled for 60-kDa mitochondrial antigen or lipid droplet-associated adipophilin. In this last case, cells transfected with COX8-mCherry were used. These various experimental approaches enabled us to conclude that both lipid droplets and mitochondria were present in extracellular lipidosomes (Fig. 8A-D).

Under conditions of oxidative stress, it was recently demonstrated in mouse fibroblasts and neutrophils that damaged mitochondria are translocated from the cell body to another type of large EVs called migrasomes, hence participating in mitocytosis, a migrasome-mediated mitochondrial quality-control process [57]. Here, we investigated whether the composition of extracellular lipidosomes were altered by the protonophore CCCP, a well-known uncoupler of mitochondrial oxidative phosphorylation. To that end, FEMX-I cells were incubated with 1 or 4 µM CCCP or without for 8 h, and then processed for CLSM. They were stained with BODIPY™ 493/503 and MitoView™ Fix 640, and then counterstained with fluorescence-conjugated WGA. Under these conditions, neither cell division nor the presence of damaged (rounded) mitochondria at the cell extremities were affected by CCCP treatment, irrespective of its concentration (Fig. 8E, data not shown). The number of cell extremities containing mitochondria remained similar to the control (DMSO) (Fig. 8F). Likewise, mitochondria stayed associated with extracellular lipidosomes in the presence of the drug. Interestingly, the number of particles released without mitochondria (containing or not lipid droplets) appeared to decrease slightly after incubation with 4 µM CCCP (Fig. 8G). Although this reduction is not significant, it could suggest that under other harsh conditions, mitochondria might be released via these particles. CCCP treatment did not impact the incorporation of lipid droplets in extracellular lipidosomes (Fig. 8G).

### Stem and cancer cell marker CD133 is found on the surface of extracellular lipidosomes

**Fig. 9:**
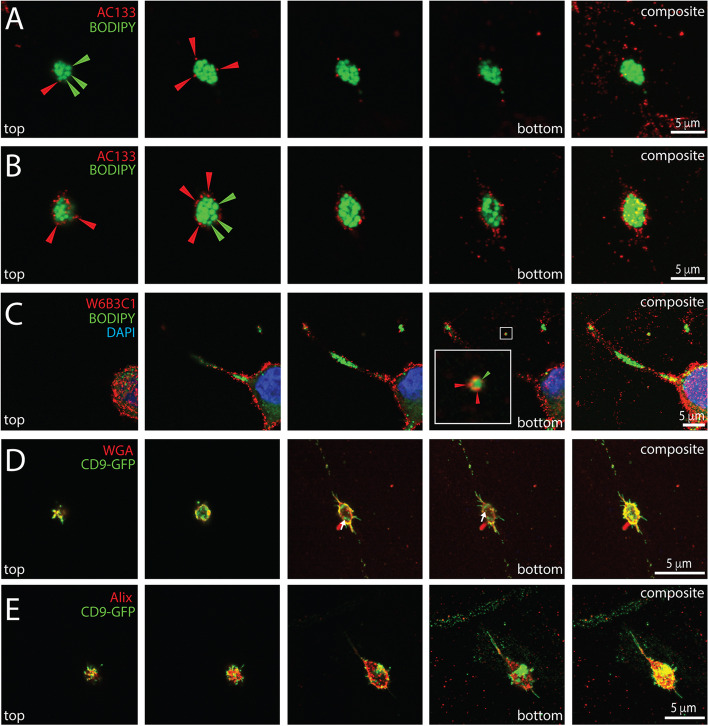
Detection of CD133 on melanoma cell-derived extracellular lipidosomes. **A**-**E** Native (**A**-**C**) or CD9-GFP-transfected (**D**, **E**) FEMX-I cells growing on poly-L-lysine coverslips were processed for CLSM. PFA-fixed cells without (**A**-**D**) or with (**E**) saponin-permeabilization were immunolabeled with an anti-CD133 antibody (clone AC133 or W6B3C1) and co-stained with BODIPY™ 493/503 (**A**-**C**). Alternatively, they were either stained with fluorescence-conjugated WGA (**D**), which labels glycoconjugates at the cell membrane, or immunolabeled for Alix (**E**) and co-observed with CD9-GFP. Cells were counterstained with DAPI to highlight nuclei. Single x–y optical sections from top to bottom (four panels on the left) and composite (right panel) images of all sections are shown. A small CD133^+^ EV containing few lipid droplets is enlarged in panel **C**. Red and green arrowheads indicate CD133 and lipid droplets, respectively, while white arrow points CD9-GFP inside the large EVs. Scale bars are indicated

The expression of CD133 on FEMX-I cells has been previously demonstrated [14], and in line with that study the cell surface CD133 immunolabeling revealed its presence in whole cells, i.e. in cell bodies and cell extremities, where lipid droplets stained with BODIPY™ 493/503 are concentrated (Additional file 1: Fig. S4A, B, inset b). In dividing cells, CD133 remained all over the cell surface, including at the extremities, suggesting that it may be a component of extracellular lipidosomes (Additional file 1: Fig. S4C). Indeed, CD133 was present on their surface, as shown by CLSM (Fig. 9A, B). Smaller extracellular lipidosomes (< 2 µm) were occasionally detected as well (Fig. 9C), which is consistent with their being released by migrating cells (see above). In line with the role of CD9 in membrane shaping (see above), CD9-GFP was also found on the surface of these large EVs (Fig. 9D). Moreover, the CD9-GFP protein was visible within these structures, suggesting the presence of endosomes that are known to be positive for this tetraspanin membrane protein [58]. ALG-2-intreacting protein X (Alix), a protein regulating endosomal trafficking and a CD9-interacting partner [59, 60] was also detected there, raising the possibility that in addition to lipid droplets and mitochondria, other types of organelles are potentially incorporated into these large EVs. This latter issue will require further investigation.

### Silencing CD133 affects the subcellular localization of lipid droplets and their incorporation in extracellular lipidosomes

**Fig. 10:**
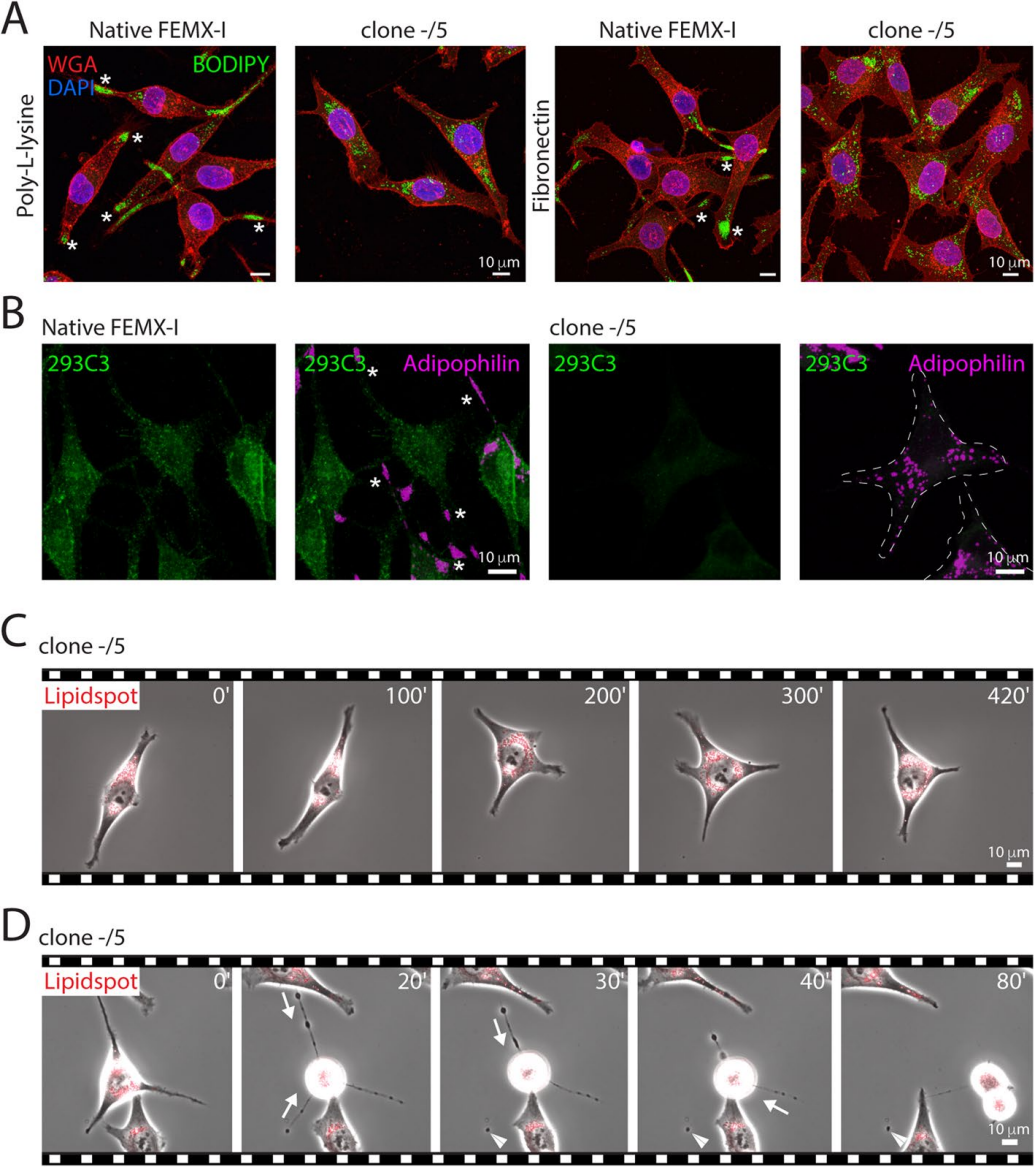
Silencing CD133 affects the distribution of lipid droplets and impedes the biogenesis of extracellular lipidosomes. **A**-**D** Native or CD133-deficient (clone –/5) FEMX-I cells growing either on poly-L-lysine (**A**) or fibronectin-coated (**A**-**D**) supports were either processed for CLSM (**A**, **B**) or recorded in live by phase-contrast/fluorescence video microscopy after staining with LipidSpot™ 610 (**C**, **D**). For CLSM, PFA-fixed cells without (**A**) or with (**B**) saponin-permeabilization were co-stained with BODIPY™ 493/503 and fluorescence-conjugated WGA (**A**) to highlight lipid droplets and glycoconjugates at the cell membrane, respectively, or double-immunolabeled with antibodies directed against CD133 (293C3) and adipophilin (**B**). For the live cell imaging, elapsed time in minutes is shown on the top-right corner. The images are excerpted from the Additional file 11: Video S10 (**C**) and Additional file 12: Video S11 (**D**). Note the EVs lacking lipid droplets during cell division of CD133-deficient cells (**D**, arrowhead). Asterisks indicate the concentration of lipid droplets at cell extremities of native cells (**A**, **B**), while arrows indicate the retraction of cell extremities (**D**). The dashed lines mark the outline of the CD133-deficient cells (**B**). Scale bars are indicated

We have previously demonstrated that silencing CD133 in melanoma cells has an impact on cell proliferation and migration as well as on the amount of lipid droplets [14, 18]. Interestingly, analysis of FEMX-I cells silenced for CD133 (clone –/5) revealed a redistribution of lipid droplets from the cell extremities towards the perinuclear region, as observed after fixation and staining with a fluorescent lipid droplet dye (Fig. 10A). This phenomenon occurred independently of the matrix substrates (Fig. 10A). The absence of CD133 and the redistribution of lipid droplets in CD133-deficient FEMX-I cells were confirmed by double immunolabeling for CD133 and adipophilin (Fig. 10B). The perinuclear localization of lipid droplets was also observed by time-lapse video microscopy of LipidSpot-stained cells, thereby ruling out potential artifacts associated with cell fixation (Fig. 10C). In contrast, the distribution of mitochondria was not affected by the absence of CD133 as they could still be observed at cell extremities (Additional file 1: Fig. S5A, B). Interestingly, the redistribution of lipid droplets from cell extremities to the perinuclear region led to the formation of large EVs depleted of lipid droplets, as observed in dividing CD133-deficient cells by video microscopy (Fig. 10D) or CLSM (Additional file 1: Fig. S5). Quantification revealed that these EVs are either completely or partially devoid of lipid droplets compared to wild-type cells (Additional file 1: Fig. S6A). EVs without lipid droplets accounted for around 5 and 20% in wild-type and CD133-deficient cells, respectively, while those containing more than 20 lipid droplets fell from 20% to zero. Likewise, smaller extracellular lipidosomes that are released by migrating cells (see above) were also lacking lipid droplets in clone –/5 cells (Additional file 1: Fig. S6B). It remains to be determined how the absence of CD133 controls the distribution of lipid droplets in cells.

### Lipid droplets are not associated with classic small CD133^+^ extracellular vesicles

In various cell systems, we have reported the association of CD133 with small EVs (e.g., exosomes and ectosomes) that are released into various body fluids or into the conditioned medium of primary stem and progenitor cells or established cell lines [61–64]. Given that melanoma FEMX-I cells also release them [18], we investigated the potential presence of lipid droplets in such small CD133^+^ EVs. To that end, FEMX-I cells were incubated for 24 h and conditioned medium was collected and processed by differential centrifugation (see “Methods” section). Each fraction and the final supernatant were analyzed by immunoblotting for CD133 and adipophilin. While both proteins were associated with the cell lysate and 400* g* pellet, which contains detached cells, only CD133 was recovered in the 10,000* g* and 200,000* g* pellets (Additional file 1: Fig. S7A). As reported before [18], the latter fraction contains small EVs such as exosomes or ectosomes, which are observed by TEM after CD133-immunogold labeling (Additional file 1: Fig. S7B). The size distribution of small CD133^+^ EVs enriched from the conditioned media was previously determined by nanoparticle tracking analysis, with an average size of about 100 nm (see Ref. [24]). Altogether, these findings suggest that lipid droplets are not associated with classic small CD133^+^ EVs released by FEMX-I cells.

## Discussion

In this study, we have described a new type of large EVs that are generated during cell division of aggressive melanoma cells. These are enriched with lipid droplets and mitochondria. Their formation is triggered by three cellular and/or molecular processes involving i) an accumulation of lipid droplets at the cell extremities; ii) a failure of the membrane extremities to retract at the mitotic entry, and iii) the presence of the cholesterol-binding membrane protein CD133, which impacts the subcellular localization of lipid droplets and their incorporation into nascent extracellular lipidosomes.

EVs have attracted considerable interest over the last decade, notably because of their role as biological nano- to micro-sized vesicles involved in the transfer of materials (e.g., proteins, membrane lipids and nucleic acids) between cells over short or long distances. Such intercellular communication plays multiple roles in physiological and pathological conditions [65]. In the case of cancer, in addition to promoting the expansion of cancer cells at the primary site, EVs participate in the development of the pre-metastatic niche, paving the way for metastatic growth at distant sites [66]. Through EVs, cancer cells interact with resident cells such as fibroblasts and mesenchymal stromal cells, vascular endothelial cells and immune cells to promote their own growth and dissemination. Different types of EV have been described, with distinct mechanisms of biogenesis (reviewed in Refs [67, 68]). These include multivesicular body-derived exosomes and ectosomes shed from the plasma membrane, both entities being less than 120–150 nm in diameter [44, 69]. Larger particles or other types of EVs have also been reported, such as apoptotic bodies (diameter 1–5 µm), released upon cell fragmentation during apoptotic cell death [70]; large oncosomes (1–10 µm), released from non-apoptotic membrane blebs of migrating cancer cells harbouring an amoeboid phenotype [71, 72] (see also Ref. [73]); migrasomes (0.5–3 µm), released upon degradation of retraction fibers left behind migrating cells or during membrane cell retraction [74, 75]; and secreted midbody remnants (200–700 nm), discarded by newly-formed sister cells after final stages of cell division [61, 76–78]. Here, we have described a novel mechanism leading to the formation of large EVs containing lipid droplets and mitochondria with diameters ranging from 2 to 6 µm. They were released during the initial stage of cell division, when cellular processes are retracting.

Although less frequent, the formation of extracellular lipidosomes was also observed during cell migration, when the trailing extremity did not fully retract. In the latter case, smaller EVs ($\le$ 2 µm) containing few lipid droplets were detected as well. In both scenarios (i.e. cell division and migration), a defect in the complete membrane retraction was responsible for the formation of these peculiar EVs. Extracellular lipidosomes thus differ from the large oncosomes produced by the membrane blebbing and shedding of invasive cancer cells. Interestingly, similar large ITG β1^+^ EVs containing lipid droplets and mitochondria have previously been described as being shed directly from the surface of astrocytes, i.e. non-cancerous cells [79]. Although the mechanism of release is distinct, it nevertheless shows the wide distribution of these new types of lipid droplet-containing EVs. Further studies are needed to classify all these large biological lipid droplet-containing entities and the mechanism(s) underlying their release.

The size of these EVs further adds to their complexity or heterogeneity, as small exosome-sized vesicles containing lipid droplets released by adipocytes and contributing to the regulation of immune cells, in particular adipose tissue macrophages, have been reported [80]. Likewise for the milk fat globules that are transported to the apical domain of the epithelial mammary secretory cells and released in milk [81]. The absence of adipophilin in CD133^+^ EVs recovered in the 200,000* g* pellet fraction upon ultracentrifugation of FEMX-I cell-derived conditioned medium, suggests that small CD133^+^ extracellular lipidosomes are also distinct from classical CD133^+^ EVs (e.g., exosomes or ectosomes release from membrane protrusions), or that they are below detection levels, consistent with their rare immunodetection by CLSM. It is particularly interesting to note that a minute fraction of CD133 immunoreactivity was observed in the supernatant at 200,000* g,* which normally contains fully soluble proteins [41], but may yet contain such vesicles given their high lipid content. Further experiments using size fractionation columns and filtration may address these issues [80].

The presence of certain ITGs that regulate cell adhesion could influence the formation of extracellular lipidosomes at the mitotic entry [82], as could certain extracellular matrix (ECM) proteins. This is particularly true for ITG αVβ3 (i.e. the Vitronectin receptor), which is concentrated at the cell extremities and lies at the contact site between extracellular lipidosomes and the ECM protein. In vivo, such an interaction between melanoma cells and Vitronectin could perhaps play a role in the release of extracellular lipidosomes, and thus stimulate lymphatic metastasis [54, 83, 84]. Similarly, ITG α2 and β1 could contribute to such processes. However, knockdown of CD9, an interacting partner of ITG β1 [46], did not prevent the formation of extracellular lipidosomes, although it did have an impact on the membrane spreading of cell extremities. A similar observation was made in breast cancer cells upon silencing of CD9 [48]. It remains to be determined whether ITGs and certain ECM proteins, notably Vitronectin, can have an action on the biogenesis of extracellular lipidosomes and/or whether the latter impact the ECM degradation.

The occurrence and/or enrichment of lipid droplets in the extracellular lipidosome is linked to their subcellular localization at the extremities of FEMX-I cells, which was confirmed by data obtained upon silencing CD133 (this study, [18]). Such subcellular localization of lipid droplets has been noted previously in primary melanoma cells, where they appeared at the cell periphery near the filopodia [85], suggesting that this phenomenon is not limited to a specific melanoma cell line. A recent study has shown a differential localization of lipid droplets among various melanoma cell lines, where the A2058 cell line showed an accumulation of lipid droplets at the cell extremities, just like FEMX-I cells, while other lines like RPMI-7951, SK-MEL-5 and SK-MEL-28 harbored lipid droplets in the perinuclear regions like in FEMX-I clone –/5 cells [23]. Although the distribution of lipid droplets was not discussed in the latter study, the classification of these melanoma cell lines as undifferentiated/neural crest (RPMI-7951), transitory (or intermediate) (A2058) and melanocytic cell (SK-MEL-5 and SK-MEL-28) states, suggests that this particular trait may be associated with a certain state of melanoma cells [86, 87]. It remains to be determined whether a correlation between subcellular localization of lipid droplets, release of extracellular lipidosomes and differentiation status exist. The same applies to the presence of CD133, which has been shown to be lost after differentiation [61–64]. As the distribution of mitochondria, as well as the expression of a given ITG (J.K., unpublished observation), remains independent of CD133 expression, this raises another question, that of the direct (or indirect) contribution of CD133 to the stability, distribution and dynamics of lipid droplets. These processes could be linked to cytoskeletal components such as microtubules and/or F-actin as described in various cellular systems and in dividing cells [88] (reviewed in Ref. [89]). Intermediate filaments could also be involved in stabilizing lipid droplets at cell extremities [90, 91], although the observation of lipid droplets accumulation at extremities of cells lacking Nestin excludes its direct involvement. However, only this intermediate filament protein is observed in the long, thin processes linking a cell and the potential extracellular lipidosome derived from it, raising the possibility that Nestin is involved in the complete retraction of cell extremities during cell division and/or migration. Overexpression of Nestin has been associated with advanced stages of melanoma, the invasion front and sites of melanoma metastases (see Ref. [92] and references therein). It will be interesting to isolate Nestin positive and negative fractions from FEMX-I cells and determine their impact on extracellular lipidosome formation, among other features [93]. It is worth noting that circulating Nestin and CD133 double-positive cancer cells have been detected in the peripheral blood of advanced melanoma patients, suggesting that both proteins may contribute to the malignant processes of melanoma [6, 94, 95].

Functionally, the enrichment of lipid droplets and mitochondria at the cell extremities may contribute in some way to membrane dynamics, and thus to the spread and/or migration of these cancer cells. Given that FEMX-I cells exhibit an aggressive metastatic trait as demonstrated after injection into immunodeficient mice [13, 14], the link between CD133 and lipid droplets/mitochondria in relation to cell migration merits further investigation. Removal of damaged mitochondria via extracellular lipidosomes, as recently reported for migrasomes [57], could be a potential function related to the release of these membrane particles under stress conditions. Although we did not observe significant increase in the discharge of large mitochondria-associated EVs upon application of mild mitochondrial stress, it cannot be excluded that mitochondria and/or other cellular contents may be eliminated under the actions of specific stressful environmental factors, including those found in the cancer microenvironment. In this context, mitochondria often appear rounded in extracellular lipidosomes, which may be due to their compaction with lipid droplets and/or the specific incorporation of damaged mitochondria. These issues need to be investigated further, particularly with regard to the influence of oxygen concentration and low pH. The release of extracellular lipidosomes may have an impact not only on donor cells, as recently shown for melanomas with increased lipid droplet capacity that were at a metabolic advantage [23], but also on the recipient cells that take them up. The mitochondria being the energy factories and the lipid droplets the energy reservoirs, the transfer of lipid droplet-mitochondrion complex might favor the metabolic activities of the recipient cells, and perhaps their transformation. Besides the bioenergetic capacity, they could reduce the β-oxidation capacity and support lipid droplet expansion. In various cancers, the accumulation of lipid droplets has been associated with tumor aggressiveness and resistance to chemotherapy [33]. Interestingly, targeting the lipid metabolism impairs resistance to BRAF kinase inhibitor in melanoma [96]. Overall, lipid droplets may have various implications in heterogeneous cell population of tumor microenvironment (reviewed in Ref. [97]). Further studies are therefore needed to determine firstly the role of CD133 (and other molecular players) in the segregation of lipid droplets in donor cells as well as in the release of extracellular lipidosomes, and secondly the impact of the latter on the biochemistry of recipient cells, particularly those associated with the cancer microenvironment. The mechanism of uptake of extracellular lipidosomes by recipient cells will also merit particular consideration, especially if specific chemokines (or other signaling molecules) are involved in attracting recipient cells toward them, as has been demonstrated for migrasomes associated with either zebrafish cells during the gastrulation or human stromal stem cells [75, 98].

In conclusion, CD133 could not only be involved in the maintenance of the stem cell state and/or actively participate in cell migration, for example during the metastasis process (reviewed in Ref. [10]), but also supply, via the novel structure described here, i.e. extracellular lipidosomes, or other types of CD133^+^ EVs, to the surrounding cellular milieu the appropriate components and/or “fuel” for their transformation to support cancer growth. Of course, these different scenarios are not mutually exclusive and may act in a sequential and orchestrated manner.

### Supplementary Information

**Additional file 1: Supplemental Figures S1-S7.**

**Additional file 2: Supplemental Video S1.** Phase contrast time-lapse video of LipidSpot™ 610-stained FEMX-I cells growing on fibronectin-coated glass-bottom dishes, depicting the concentration of lipid droplets at the cell extremities and its symmetric division between the two nascent extremities. The elapsed time in minutes is shown on the top-right corner. Still images from this video are shown in both Fig. 2B and Additional file 1: Fig. S3A.

**Additional file 3: Supplemental Video S2.** Phase contrast time-lapse video of LipidSpot™ 610-stained FEMX-I cells growing on fibronectin-coated glass-bottom dishes, depicting the asymmetric distribution and translocation of lipid droplets between the two nascent extremities. The elapsed time in minutes is shown on the top-right corner. Still images from this video are shown in Additional file 1: Fig. S3B.

**Additional file 4: Supplemental Video S3.** Phase contrast time-lapse video of LipidSpot™ 488-stained FEMX-I cells growing on fibronectin-coated glass-bottom dishes, depicting the resorption of lipid droplets from their extremities. The elapsed time in minutes is shown on the top-right corner. Still images from this video are shown in Fig. 4A.

**Additional file 5: Supplemental Video S4.** Phase contrast time-lapse video of LipidSpot™ 488-stained FEMX-I cells growing on fibronectin-coated glass-bottom dishes, depicting the resorption of lipid droplets from their extremities. The elapsed time in minutes is shown on the top-right corner.

**Additional file 6: Supplemental Video S5.** Phase contrast time-lapse video of LipidSpot™ 488-stained FEMX-I cells growing on fibronectin-coated glass-bottom dishes, depicting the release of an extracellular lipidosome during cell division. The elapsed time in minutes is shown on the top-right corner. Still images from this video are shown in Fig. 4B.

**Additional file 7: Supplemental Video S6.** Phase contrast time-lapse video of LipidSpot™ 610-stained FEMX-I cells growing on fibronectin-coated glass-bottom dishes, depicting the release of a large extracellular lipidosome from a cell extremity during cell migration. The elapsed time in minutes is shown on the top-right corner. Still images from this video are shown in Fig. 4C.

**Additional file 8: Supplemental Video S7.** Phase contrast time-lapse video of LipidSpot™ 610-stained FEMX-I cells growing on fibronectin-coated glass-bottom dishes, depicting the release of a small extracellular lipidosome from a cell extremity during cell migration. The elapsed time in minutes is shown on the top-right corner. Still images from this video are shown in Fig. 4D.

**Additional file 9: Supplemental Video S8.** Phase contrast time-lapse video of LipidSpot™ 610-stained FEMX-I cells growing on fibronectin-coated glass-bottom dishes, depicting the uptake of an extracellular lipidosome (arrow) by FEMX-I cells. The elapsed time in minutes is shown on the top-right corner. Still images from this video are shown in Fig. 5A.

**Additional file 10: Supplemental Video S9.** Phase contrast time-lapse video of LipidSpot™ 610-stained FEMX-I cells growing on fibronectin-coated glass-bottom dishes, depicting the uptake of an extracellular lipidosome. The elapsed time in minutes is shown on the top-right corner. Still images from this video are shown in Fig. 5B.

**Additional file 11: Supplemental Video S10.** Phase contrast time-lapse video of LipidSpot™ 610-stained FEMX-I clone –/5 cells growing on fibronectin-coated glass-bottom dishes, depicting the distribution of lipid droplets at the perinuclear region. The elapsed time in minutes is shown on the top-right corner. Still images from this video are shown in Fig. 10B.

**Additional file 12: Supplemental Video S11**. Phase contrast time-lapse video of LipidSpot™ 610-stained FEMX-I clone –/5 cells growing on fibronectin-coated glass-bottom dishes, depicting the release of a lipid droplet-free EV. The elapsed time in minutes is shown on the top-right corner. Still images from this video are shown in Fig. 10C.

## Data Availability

No datasets were generated or analysed during the current study.

## Abbreviations

Alix: ALG-2-intreacting protein X
APC: Allophycocyanin
CCCP: Carbonyl cyanide m-chlorophenyl hydrazine
CLSM: Confocal laser-scanning microscopy
DMSO: Dimethyl sulfoxide
ECM: Extracellular matrix
EV: Extracellular vesicle
GFP: Green fluorescent protein
ITG: Integrin
MFI: Median fluorescence intensity
PBS: Phosphate-buffered saline
PE: Phycoerythrin
PFA: Paraformaldehyde
SEM: Scanning electron microscopy
SDS: Sodium dodecyl sulfate
TEM: Transmission electron microscopy
WGA: Wheat germ agglutinin
