# Supplementary material for: Extracellular lipidosomes containing lipid droplets and mitochondria are released during melanoma cell division
Source: Cell Commun Signal. 2024 Jan 19;22:57. doi: 10.1186/s12964-024-01471-7 (PMC10799373; doi:10.1186/s12964-024-01471-7)
Supplement: Supplementary file 1 — Additional file 1: Supplemental Figures S1-S7. [file 12964_2024_1471_MOESM1_ESM.docx]

**Supplementary Information**

**Extracellular lipidosomes containing lipid droplets and mitochondria are released during melanoma cell division**

Jana Karbanová, Ilker A. Deniz, Michaela Wilsch-Bräuninger, Rita Alexandra de Sousa Couto, Christine A. Fargeas, Mark F. Santos, Aurelio Lorico and Denis Corbeil

**Additional file 1:** Supplemental Figures S1-S7


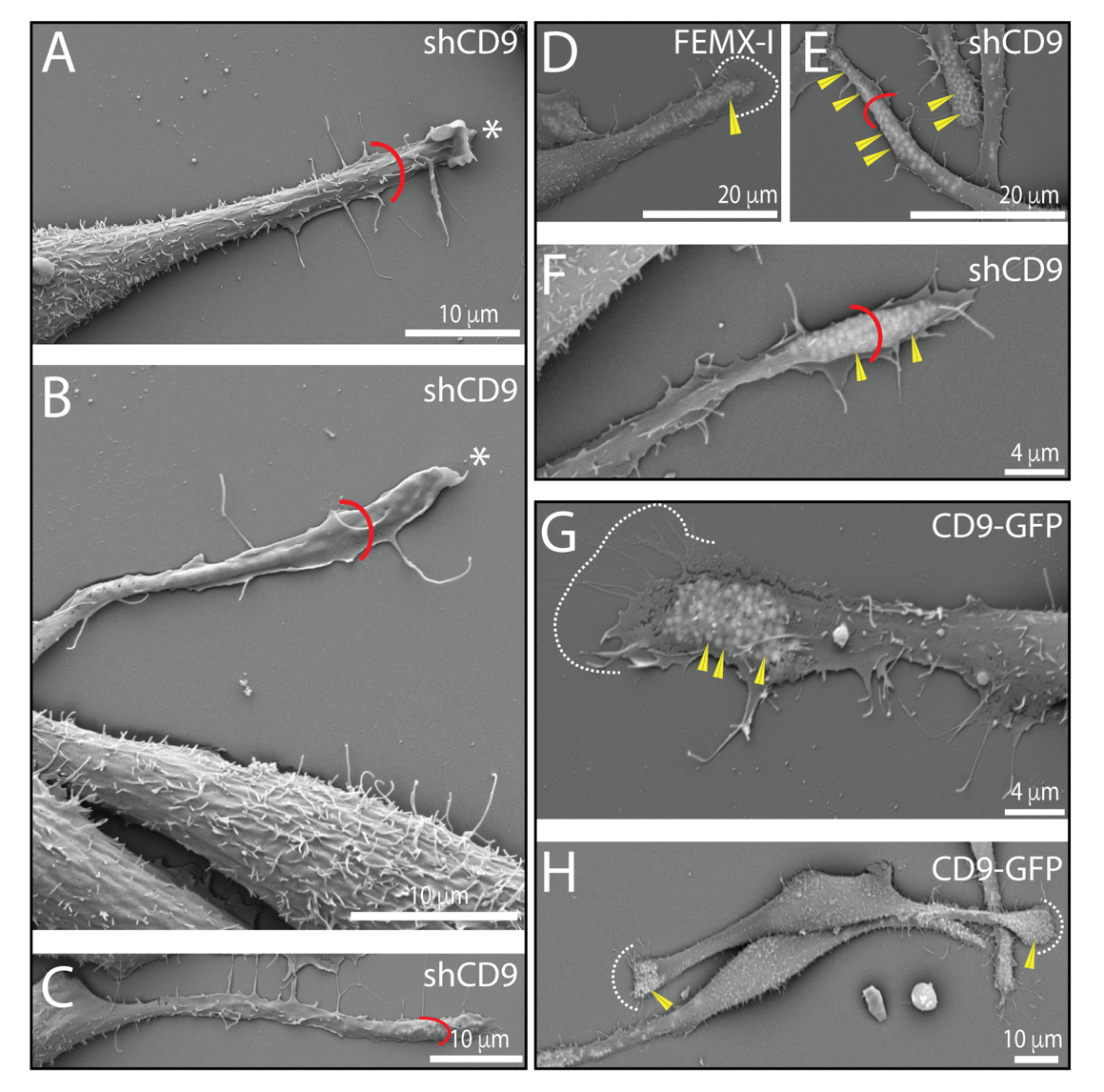
**Supplemental Figures**

**Fig. S1** CD9 affects the morphology of the extremities of FEMX-I cells, but not the accumulation of lipid droplets within them. **A**-**H** FEMX-I cells (**D**) and their counterparts lacking CD9 (shCD9, **A-C**, **E**, **F**) or overexpressing CD9-GFP (CD9-GFP, **G**, **H**) were grown on poly-L-lysine-coated coverslips and analyzed by SEM. Note that extremities are narrow in CD9-deficient cells (**A-C**, **E**, **F**, red arc and asterisk) compared to parental cells (**D**) and those expressing GFP fusion protein (**G**, **H**), where their extremities are flattened with filopodia adhering to the support (dashed line). Lipid droplets were detected in these extremities by applying higher accelerating voltage, independently of the CD9 expression (**D-H**, yellow arrowhead). Scale bars are indicated.


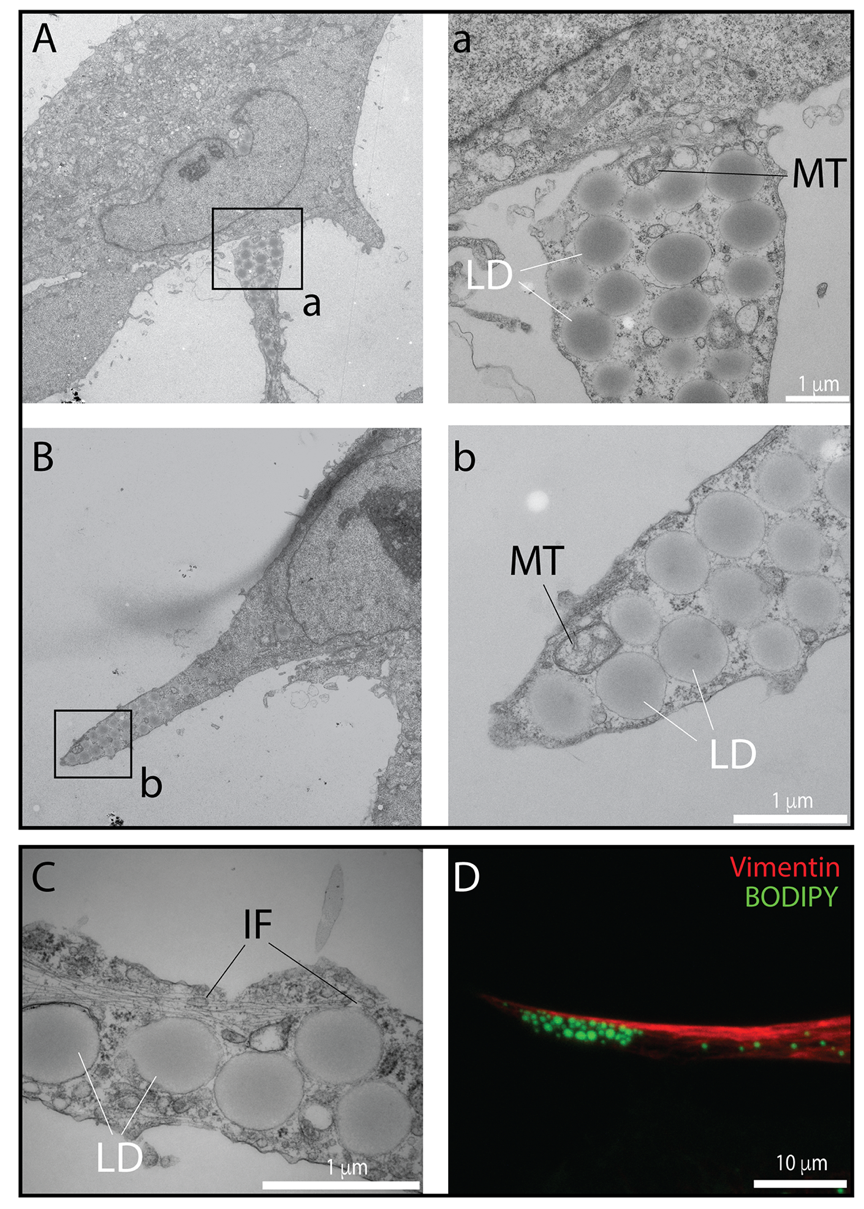


**Fig. S2** Lipid droplets are found in the proximity of mitochondria and intermediate filaments. **A-D** FEMX-I cells growing on plastic Petri dishes (**A-C**) or poly-L-lysine-coated coverslips (**D**) were processed for TEM (**A-C**) and CLSM (**D**). For the latter, PFA-fixed cells were saponin-permeabilized, immunolabeled for Vimentin and stained with BODIPY™ 493/503. Subpanels a and b are enlargements of the area boxed in **A** and **B**, respectively. Note the proximity of the lipid droplets (LD) to the mitochondria (MT) and intermediate filaments (IF) at the cellular extremity (**A-C**). IF were confirmed by Vimentin immunolabeling (**D**). Scale bars are indicated.


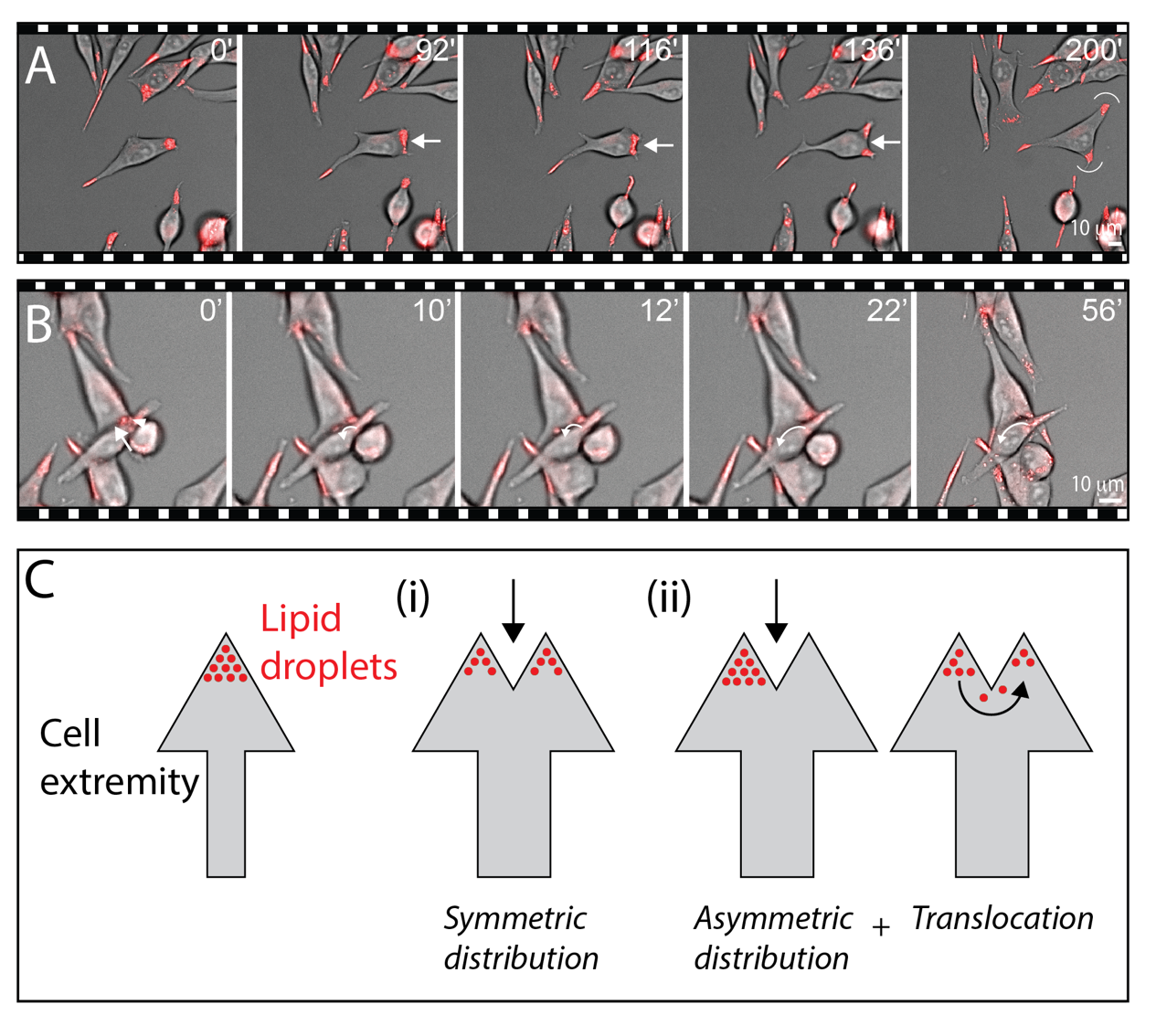


**Fig. S3** Differential distribution of lipid droplets in the dividing cell extremities. **A**, **B** FEMX-I cells growing on fibronectin were recorded by phase-contrast/fluorescent live video microscopy after staining with LipidSpot™ 610. Lipid droplets accumulated at the cell extremities are distributed symmetrically (**A**) or asymmetrically (**B**) upon cell extremity partition (white arrow). A redistribution of lipid droplets between the new extremities is observed in the latter case. Elapsed time in minutes is shown in the top-right corner. The images are excerpted from the Additional file 2:Video S1 (**A**) and Additional file 3:Video S2 (**B**). (**C**) Schematic illustration of lipid droplets distribution during cell extremity partition. Scale bars are indicated.


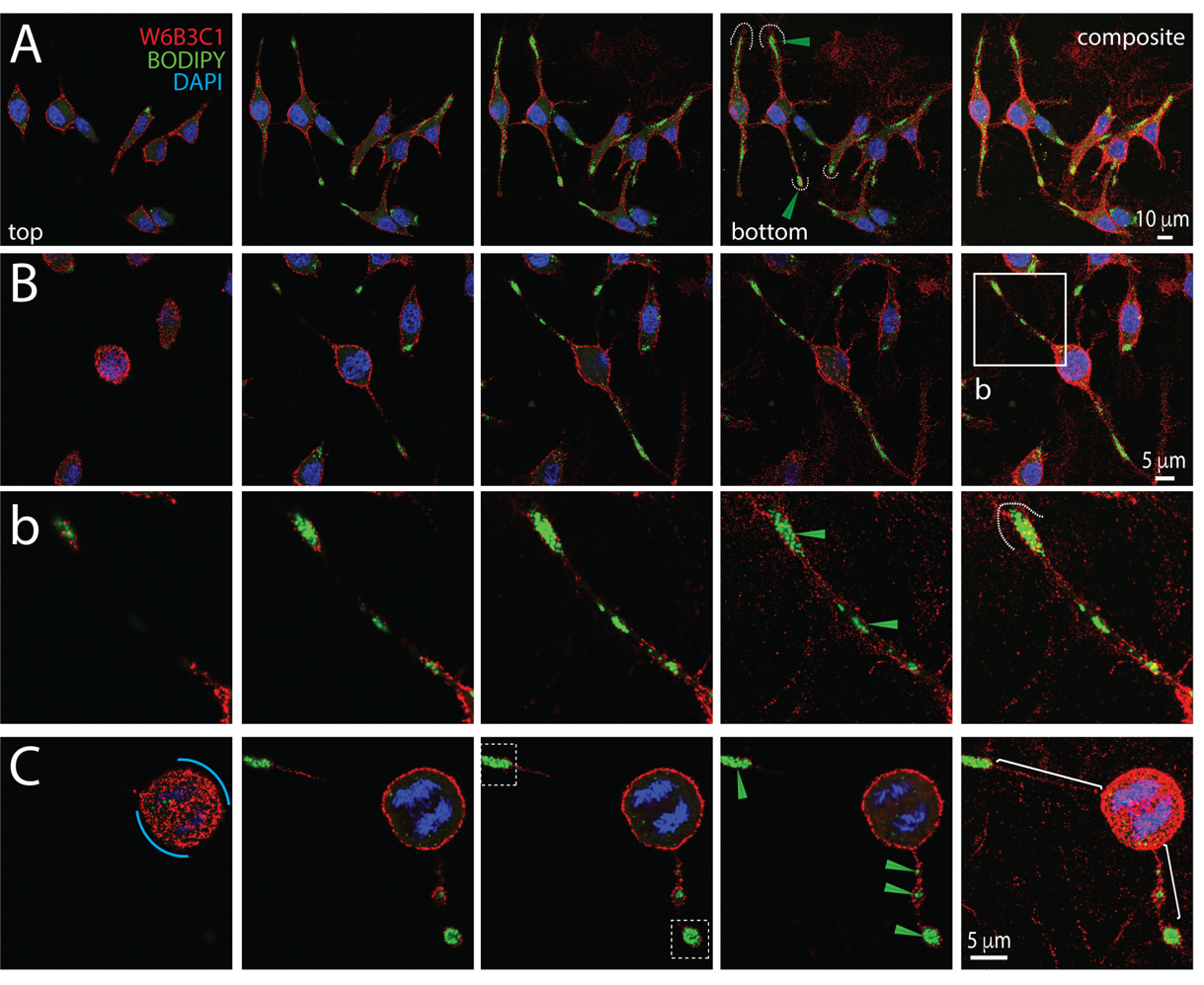


**Fig. S4** Cell surface expression of CD133 on melanoma cells. **A**-**C** FEMX-I cells growing on poly-L-lysine-coated coverslips were processed for CLSM. PFA-fixed cells without permeabilization were immunolabeled with an anti-CD133 antibody (clone W6B3C1) and stained with BODIPY™ 493/503. Cells were counterstained with DAPI to visualize nuclei. Three distinct examples are shown (**A-C**), and the boxed area in **B** indicates an enlarged region displayed in subpanel b. Single x-y optical sections from top to bottom (four panels on the left) and composite images of all sections are shown (right panel). Arrowheads indicate BODIPY^+^ lipid droplets at cell extremities (**A**, **B**) and along the thin retracting membrane (**C**, bracket) observed during cell division (blue arc). Flattened extremities with filopodia adhering to the support (**A**, **B**, dotted line) and the remaining cell extremities during cell division (**C**, dashed boxes) are indicated. Scale bars are indicated.

**
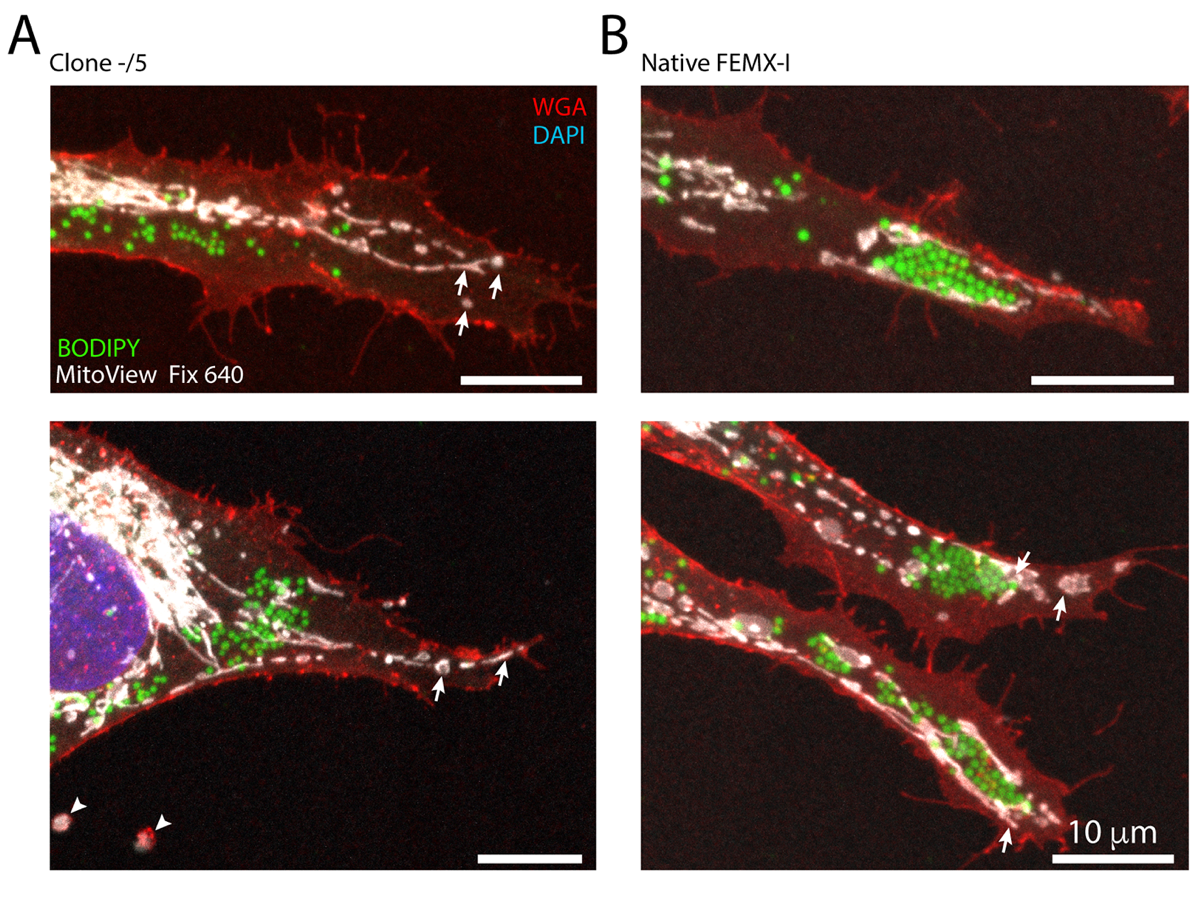
**

**Fig. S5** Silencing CD133 does not affect the distribution of mitochondria. **A, B** CD133-deficient (clone –/5, **A**) or native (**B**) FEMX-I cells growing on fibronectin-coated supports were processed for CLSM after fixation with PFA and co-staining with BODIPY™ 493/503 and MitoView^TM^ Fix 640, which highlight lipid droplets and mitochondria, respectively. Cells were counterstained with fluorescence-conjugated WGA and DAPI to visualize the glycoconjugates at the cell membrane and nuclei, respectively. Composite images of all x-y optical sections are shown. Arrows indicate mitochondria at cell extremities. Note that large EVs without lipid droplets were observed in CD133-deficient cells (**A**, arrowheads). Scale bars are indicated.


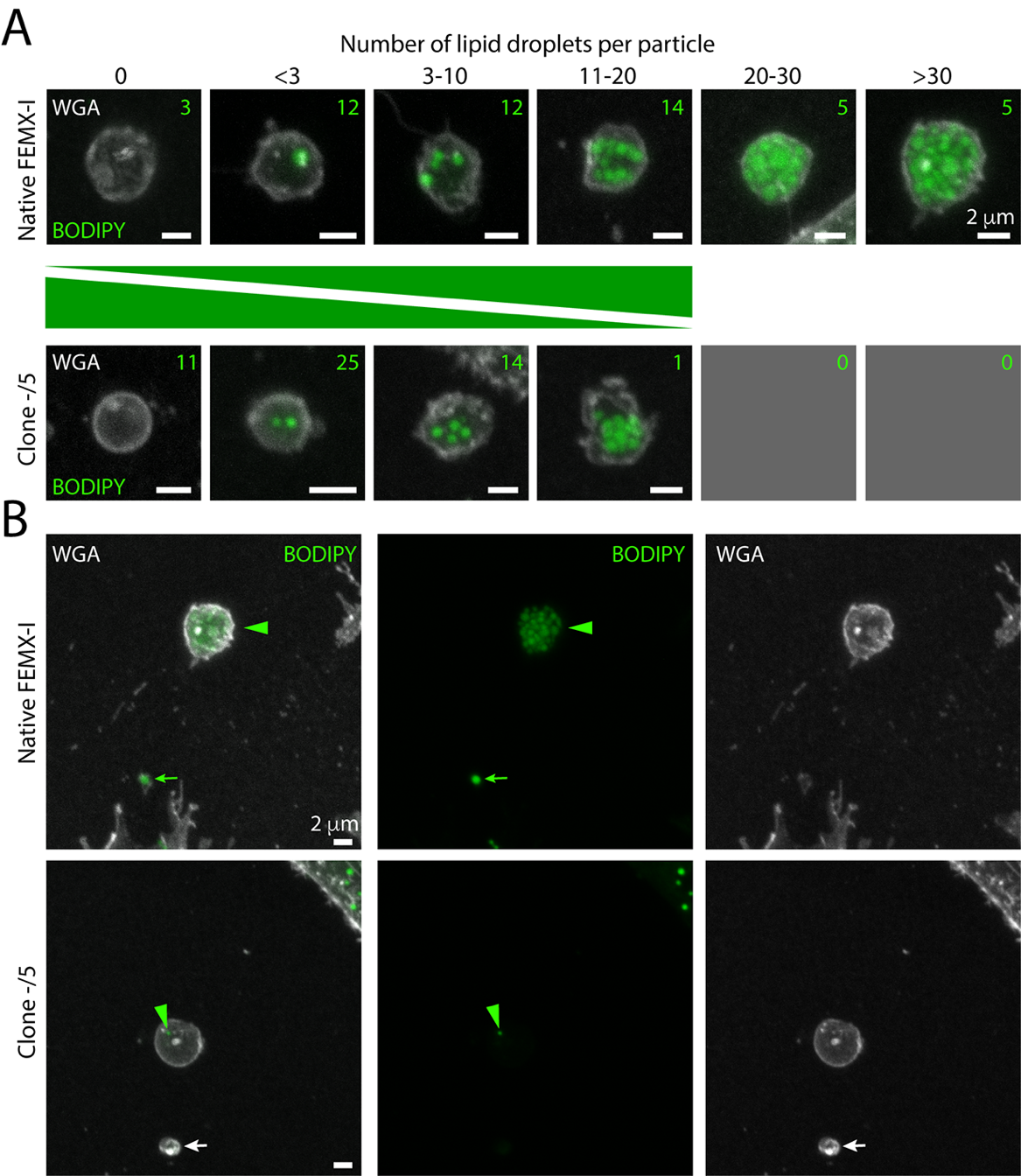


**Fig. S6** Quantification of lipid droplets in extracellular lipidosomes derived from CD133-deficient cells. **A, B** Native (top panels) and CD133-deficient (clone –/5, bottom panels) FEMX-I cells growing on fibronectin-coated supports were processed for CLSM after fixation with PFA and co-staining with BODIPY™ 493/503 and fluorescence-conjugated WGA to highlight lipid droplets and glycoconjugates at the cell membrane, respectively. Large (**A**, **B**) and small (**B**) extracellular lipidosomes derived from both cell lines were imaged, and composite images of all x-y optical sections are shown. The number of extracellular lipidosomes containing a given number of lipid droplets as indicated is shown in the top right-hand panel (**A**, n = 51 vesicles). Note the increased number of EVs without lipid droplets and the absence of extracellular lipidosomes containing more than 20 lipid droplets in CD133-deficient cells. The presence of large (arrowhead) and small (arrow) extracellular lipidosomes with few (green) or no (white) lipid droplets, respectively, in CD133-deficient cells is indicated (**B**). Scale bars are indicated.

**
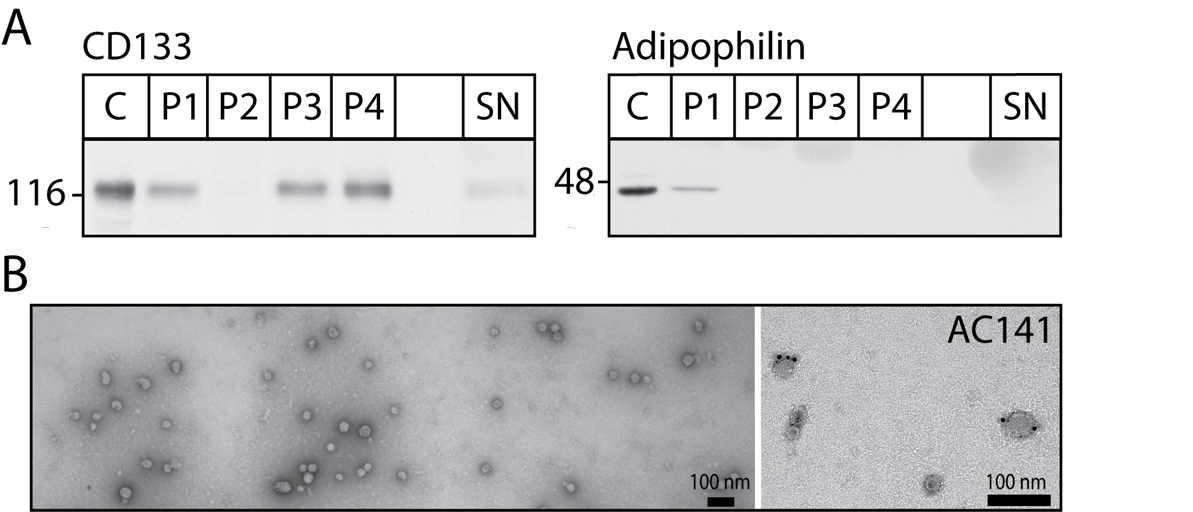
**

**Fig. S7** Lipid droplets are not associated with small CD133^+^ EVs. **A** FEMX-I cells were cultured for 24 hours and conditioned media were collected and subjected to differential centrifugation; 5 minutes at 400 x *g* (P1), 20 minutes at 1200 x *g* (P2), 30 minutes at 10,000 x *g* (P3) and 1 hour at 200,000 x *g* (P4). Proteins in the 200,000 *g* supernatants (SN) were recovered by precipitation. In parallel, detergent cell lysates (C) were prepared. The entire pellets of each fraction, and 1/10 of cell lysates or final supernatants were analyzed by immunoblotting using monoclonal antibodies against CD133 (clone 80B258, left panel) and adipophilin (AP125, right panel). Molecular mass markers (kDa) are indicated. **B** EVs recovered in the 200,000 *g* pellet (P4) were processed for TEM after immunogold labeling using a monoclonal antibody against CD133 (AC141, Miltenyi Biotec GmbH, Germany). The latter was detected using anti-mouse IgG coupled to 10-nm gold particles (British Biocell Int., UK). For details of the methods, see Ref. [18]. Note the absence of adipophilin in the P4 fraction (A), which contains small CD133^+^ EVs such as exosomes and ectosomes (B), and the unusual presence of CD133 in the 200,000 *g* supernatants. Scale bars are indicated.
